# Biguanide-Based Synthesis of 1,3,5-Triazine Derivatives with Anticancer Activity and 1,3,5-Triazine Incorporated Calcium Citrate Nanoparticles

**DOI:** 10.3390/molecules26041028

**Published:** 2021-02-15

**Authors:** Monnaya Chalermnon, Sarocha Cherdchom, Amornpun Sereemaspun, Rojrit Rojanathanes, Tanatorn Khotavivattana

**Affiliations:** 1 Department of Chemistry, Faculty of Science, Chulalongkorn University, Bangkok 10330, Thailand; monnayach@yahoo.com; 2 Department of Pharmacy Practice, Faculty of Pharmaceutical Sciences, Chulalongkorn University, Phayathai Road, Wangmai, Patumwan, Bangkok 10330, Thailand; sarocha.cherdchom@gmail.com; 3 NanoMedicine Research Unit, Department of Anatomy, Faculty of Medicine, Chulalongkorn University, Rama 4 Road, Patumwan, Bangkok 10330, Thailand; 4 Chula Medical Innovation Centre (CMIC), Nanomedicine Research Unit, Department of Anatomy, Faculty of Medicine, Chulalongkorn University, Bangkok 10330, Thailand; amornpun.s@gmail.com; 5 Centre of Excellence in Materials and Bio-Interfaces, Faculty of Science, Chulalongkorn University, Bangkok 10330, Thailand; rojrit@hotmail.com; 6 Centre of Excellence in Natural Products Chemistry, Department of Chemistry, Faculty of Science, Chulalongkorn University, Bangkok 10330, Thailand

**Keywords:** 1,3,5-triazine, anticancer, calcium citrate, nanoparticles

## Abstract

Twelve derivatives of biguanide-derived 1,3,5-triazines, a promising class of anticancer agent, were synthesised and evaluated for their anticancer activity against two colorectal cancer cell lines—HCT116 and SW620. **2c** and **3c** which are the derivatives containing *o*-hydroxyphenyl substituents exhibited the highest activity with IC_50_ against both cell lines in the range of 20–27 µM, which is comparable to the IC_50_ of cisplatin reference. Moreover, the potential use of the calcium citrate nanoparticles (CaCit NPs) as a platform for drug delivery system was studied on a selected 1,3,5-triazine derivative **2a**. Condition optimisation revealed that the source of citrate ions and reaction time significantly influence the morphology, size and %drug loading of the particles. With the optimised conditions, “CaCit-**2a** NPs” were successfully synthesised with the size of 148 ± 23 nm and %drug loading of up to 16.3%. Furthermore, it was found that the release of **2a** from the synthesised CaCit-**2a** NPs is pH-responsive, and **2a** could be control released under the acidic cancer environment. The knowledge from this study is perceptive for further development of the 1,3,5-triazine-based anticancer drugs and provide the platform for the incorporation of other drugs in the CaCit NPs in the future.

## 1. Introduction

Cancer is a devastating disease with an increasing number of diagnosis and death every year. In 2019, there were more than 1 million new cases, especially prostate, breast, lungs and colorectal cancer [1]. Chemotherapy is one of the most frequently used treatment methods despite several limitation of the currently available drugs including poor drug selectivity, potential drug resistance and possibilities for recurrences [2]. In order to mitigate these limitations, the development of a new anticancer agent with an efficient delivery system is necessary [3]. The 1,3,5-triazine scaffold has been reported to possess many biological activities especially anticancer activity [4]. A variety of synthetic routes exists for the 1,3,5-triazine derivatives, for example, three-component methods [5,6] or reactions between biguanides with alcohol [7,8], amide [9] or carboxylic acid derivatives [10,11]. As seen in many literatures, a biguanide like metformin has been frequently employed as the starting material [8,10,12,13]. Metformin-derived 1,3,5-triazine derivative HL010183 was shown to be 100 times more cytotoxic than metformin with IC_50_ of 0.28 mM for both MDA-MB-231 and Hs578T cell lines [14]. 1,3,5-Triazine synthesised from various other aryl-biguanides were also reported as Rad6 ubiquitin-conjugating enzymes inhibitors and they exhibited anticancer activity in the low micromolar range [15]. It is therefore very intriguing to explore new structural modifications of 1,3,5-triazine derivatives to further enhance the anticancer activity of this scaffold. However, one limitation of 1,3,5-triazine is its ability to reach the target site which would affect its anticancer activity [4]. In 2016, one example of the drug delivery system (DDS) was reported by coupling the 1,3,5-triazine derivatives with bovine serum albumin in order to solve this issue [4].

DDS is a safe and efficient way to deliver drug while enhancing the therapeutic efficiency and reducing side effects. Nanoparticles acting as drug carriers could benefit from the enhanced permeability and retention (EPR) effect because cancer cells have leaky vasculatures and an impaired lymphatic system [16]. One influential aspect of the EPR effect is particles sized 30–300 nm, which has been reported as a suitable range. Particle size outside of this range are prone to rapid clearance by the mononuclear phagocyte system [17,18]. Additionally, the leakiness of tumour vessel could dictate the selectivity of nanoparticles towards cancer cells. Another prominent characteristic of cancer cells is their relatively lower pH environment compared to normal cells, therefore pH-responsive nanoparticles would further reinforce the usefulness of nanoparticles for drug delivery system [19]. Recently, our group has shown a potential use of calcium citrate nanoparticles, a biocompatible and biodegradable material [20,21], as a promising platform for DDS. Vancomycin, an antibiotic drug for bone spacer application, was successfully encapsulated in the calcium citrate particles (VAN-CC) with a size of around 500 nm and an encapsulation efficiency of 28% [22]. In another work, FITC, a fluorescent agent, was encapsulated and slowly released from calcium citrate nanoparticles as experimented with human keratinocytes [23].

**Figure 1:**
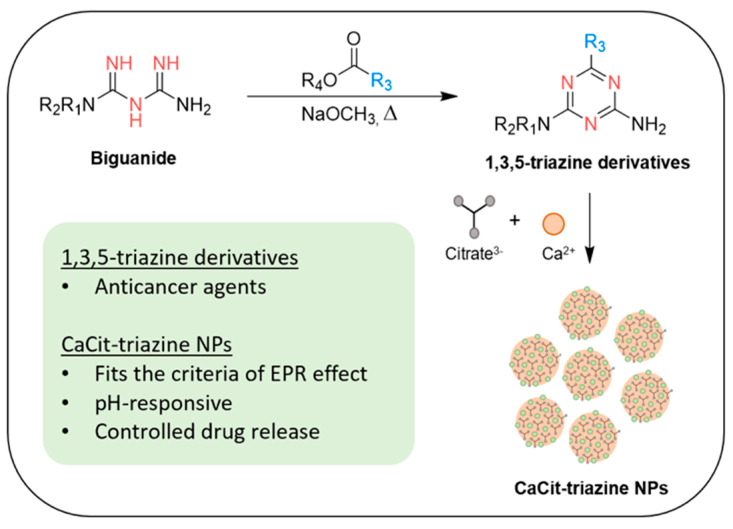
Synthesis and incorporation of biguanide-derived 1,3,5-triazine derivatives in calcium citrate nanoparticles (this work).

Herein, we report the synthesis and anticancer activities of biguanide-derived 1,3,5-triazine derivatives bearing various substituents on the triazine scaffold. Furthermore, the DDS based on the incorporation of a selected 1,3,5-triazine derivative into calcium citrate nanoparticles was investigated (Figure 1). We hypothesised that the nanoparticles would benefit from the EPR effect as well as be pH-responsive and control released drug under the acidic cancer environment.

## 2. Results and Discussions

### 2.1. Chemistry

**Figure 2:**
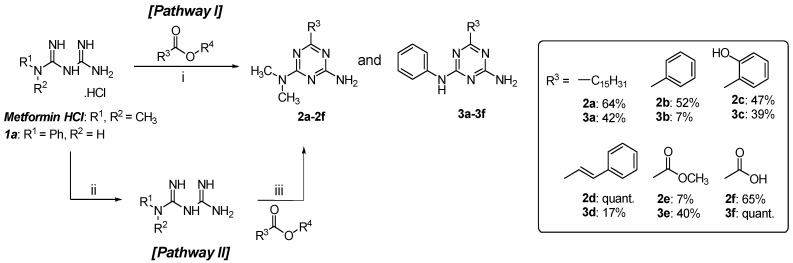
Schematic diagram of the reaction of biguanides with several esters. Reagents and conditions: (**i**) Ester (1–3 equiv.), NaOMe (1–5 equiv.), anh. MeOH, reflux; (**ii**) NaOMe (1 equiv.), anh. MeOH, rt.; (**iii**) ester (1-excess equiv.), anh. MeOH.

1,3,5-Triazine derivatives of metformin (**2a**–**2f**) and phenylbiguanide (**3a**–**3f**) were synthesised by the condensation between a biguanide and an ester. Metformin hydrochloride was used as purchased, but phenylbiguanide hydrochloride (**1a**) was prepared according to the previously reported method using aniline and dicyandiamide in excellent yield [24]. The five esters included in this work were isopropyl palmitate (R_3_ = C_15_H_31_), methyl benzoate (R_3_ = Ph), methyl salicylate (R_3_ = Ph-*o*-OH), methyl cinnamate (R_3_ = C=CH-Ph) and diethyl oxalate (R_3_ = COOC_2_H_5_). Initially, a one-pot reaction containing a biguanide hydrochloride, an ester and sodium methoxide (NaOMe) in methanol was used [10,15,24,25]. After reaction optimisation by adjusting the ratio of reagents and reaction time, **2a**, **2b**, **2d**, **2f** and **3a** were obtained in moderate yields (Figure 2, Pathway I). However, the synthetic yields of other derivatives were very low. Carboxylic by-products of the ester starting materials were detected even though anhydrous solvent and inert atmosphere were implemented. We hypothesised that this could be due to the presence of base and water in the reaction mixture simultaneously. Therefore, the method was slightly adjusted by starting with the neutralisation step to generate biguanide in the free base form [10,15,24,25]. Then the biguanide was isolated and reacted with an ester without the use of the additional base (Figure 2, Pathway II). As a result, **2c**, **2e** and **3b**–**3f** were successfully synthesised with low to moderate yields. In total, 12 1,3,5-triazine derivatives were obtained, of which four compounds were novel (**2e**, **2f**, **3d**, **3f**); three compounds have been reported, but not fully characterised (**2c**, **3a**, **3c**); and five compounds were known (**2a**, **2b**, **2d**, **3b**, **3e**). The structures of all 1,3,5-triazine derivatives were elucidated by ^1^H and ^13^C-NMR spectroscopy. In addition, the novel and not fully characterised derivatives were further characterised for IR spectra, mass spectra and melting points as the information has yet been reported (see Supplementary Materials).

### 2.2. In Vitro Anticancer Activities

The in vitro anticancer activities of **2a**–**2f** and **3a**–**3f** were evaluated against two colon cancer cell lines to assess the influence of synthesised 1,3,5-triazine derivatives on cellular viability (Table 1). Anticancer studies of 1,3,5-triazine derivatives is extensive, but only a few studies had performed their evaluations with HCT116 and SW620 cell lines [26,27]. Cisplatin was used as a positive control and the result was consistent with reported literature value [28]. Initially, a preliminary screening of the cytotoxicity was performed on all twelve 1,3,5-triazine derivatives at a specific concentration of 100 µM. All of the synthesised 1,3,5-triazine derivatives were active at varied abilities, and some exhibited comparable or higher cytotoxicity than cisplatin for both cell lines. The comparison of the cytotoxicity of these compounds at 100 µM showed that the hydrophobic substituents resulted in higher cytotoxicity than hydrophilic groups as reflected in the two most cytotoxic compounds: **2a** with the long alkyl substituent (%cytotoxicity = 96.02 ± 0.06 (SW620) and 67.43 ± 1.82 (HCT116)) and **3d** with the aryl group (87.47 ± 1.54 (SW620) and 92.27 ± 0.04 (HCT116)). In contrast, the %cytotoxicity of **2b** with phenyl substituent, **2e**/**3e** with the ester substituent and **2f**/**3f** with the carboxylic substituents were very low in the range of 5–20%. Overall, the 1,3,5-triazine that are derived from the phenylbiguanide (**3a**–**3f**) showed significantly higher cytotoxicity compared with ones derived from the metformin (**2a**–**2f**). These results established a relationship between the increased hydrophobicity with enhanced cytotoxicity, but the reason for this relation do need further investigation. From the %cytotoxicity values, **2a** was selected for the further study on the drug incorporation using calcium citrate nanoparticles.

Six compounds with high %cytotoxicity were selected for further evaluation by varying the concentration between 0–100 µM to calculate the 50% cell-growth inhibition (IC_50_) values. (see Supplementary Materials) The most potent anticancer agents were **2c** and **3c**, both having the *o*-hydroxyphenyl substituent with the IC_50_ of 25.25 ± 1.12 µM and 22.80 ± 1.06 µM for SW620 cell line, and at 26.29 ± 1.07 µM and 20.79 ± 1.07 µM for HCT116 cell line, respectively. These IC_50_ values of **2c**/**3c** were comparable and slightly better than the reference drug, cisplatin. Even though the synthesised compounds are yet novel, the activity of **2c** or **3c** compared to the previously reported 1,3,5-triazine derivatives with alike substituents and size, for example, HL010183 or 4-amino-6-(arylamino)-*N*-phenyl-1,3,5-triazine-2-carbohydrazides, could be considered as decent competitors [14,29].

### 2.3. 1,3,5-Triazine Incorporated Calcium Citrate Nanoparticles

**Figure 3:**
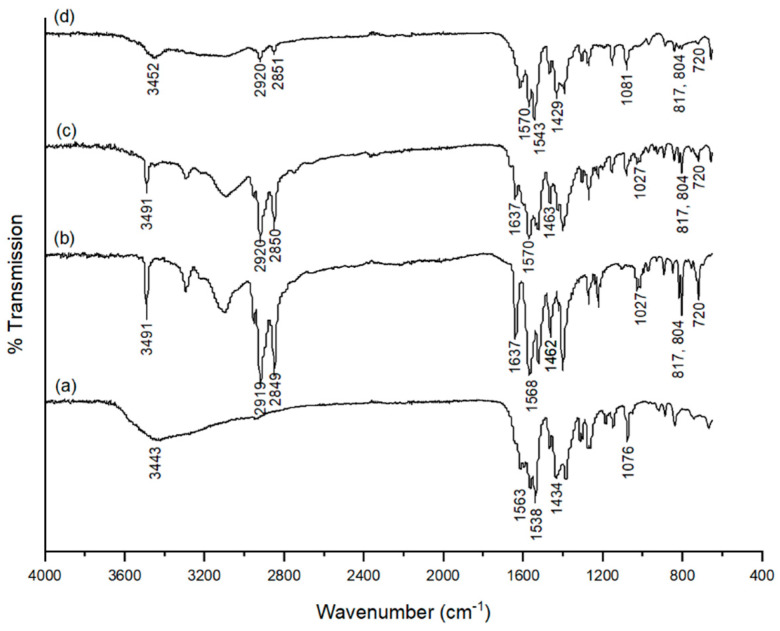
FTIR spectra of (**a**) calcium citrate nanoparticles, (**b**) **2a**, (**c**) reaction using sodium citrate and (**d**) reaction using citric acid with NaOH.

Despite the successful drug encapsulation in calcium citrate nanoparticles [23], the method was not applicable for the incorporation of **2a**. This was due to the use of water as the only solvent in the system, leading to poor solubility of triazine drugs. Therefore, we attempted to optimise the protocol for the preparation of 1,3,5-triazines-incorporated calcium citrate nanoparticles (CaCit-triazine NPs) using EtOH-water as a binary solvent system, adjusted from the synthesis of calcium carbonate particles previously reported [30]. There are many methods to prepare calcium citrate particles, but the two most common reagents are CaCl_2_ and sodium citrate [21,23,31]. In our first attempt, a solution of CaCl_2_ in EtOH was slowly added to a solution of **2a** and sodium citrate in EtOH-water under vigorous stirring at room temperature. Unfortunately, the obtained product was large precipitate particles of **2a** as confirmed by SEM and FTIR, and the desired CaCit-triazine NPs was not observed, since there was no IR signal characteristic to calcium citrate, i.e., 3400 cm^−1^ O-H stretching or peaks in the carbonyl group stretching region (Figure 3c). This was due to the basicity of **2a** and sodium citrate [32]. In alkali condition, calcium ions have more affinity towards hydroxide than citrate ions, thus allowing the possibility to form calcium hydroxide instead of calcium citrate precipitate [33]. Alternatively, calcium citrate could be prepared via acid-base reaction between citric acid and calcium hydroxide or calcium carbonate [34]. We decided to generate citrate ions in situ from the reaction between citric acid and NaOH preventing the formation of calcium hydroxide in elevated pH. The pH of citric acid solution was adjusted to pH 6.0 using 1:2 mole ratio of citric acid to NaOH resulting in citrate ions in the divalent form [35]. Both the selected pH value and the divalent form of citrate ions have been reported to allow the formation of calcium citrate [32]. The FTIR spectrum of product obtained from using citric acid with NaOH exhibited characteristic peaks of both calcium citrate (3452, 1543, 1429 and 1081 cm^−1^) and **2a** (2920, 2851, 1570, 817, 804 and 720 cm^−1^) as depicted in Figure 3d. Even though citric acid with NaOH could perform as the source of citrate, the obtained particles were aggregated and not nano-sized. Further reaction optimisation continued with the study of the effect of reaction time.

**Figure 4:**
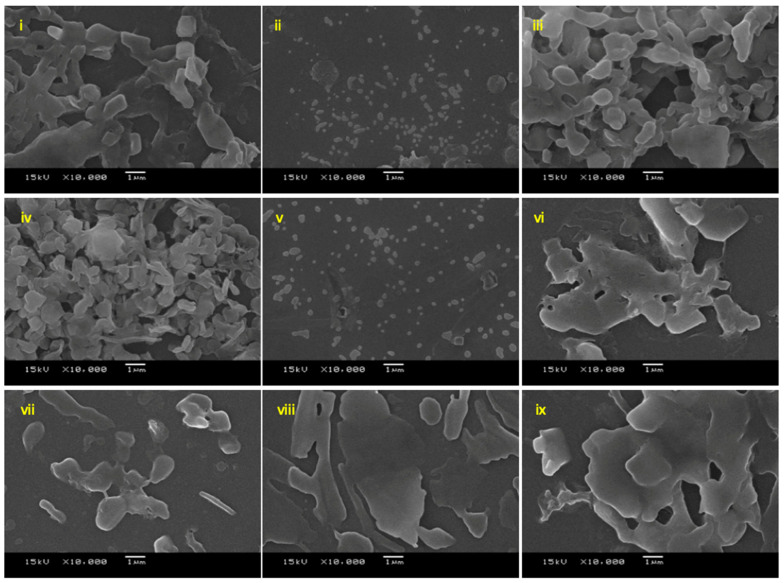
SEM images of CaCit-triazine at various synthesis time between 1–24 h at 10,000× magnification; **i** (1 h), **ii** (2 h), **iii** (4 h), **iv** (6 h), **v** (8 h), **vi** (12 h), **vii** (16 h), **viii** (20 h) and **ix** (24 h).

Reaction time significantly influence the morphology and size of particles. SEM images in Figure 4 show a general trend that particle grew larger with longer reaction time. Iafisco et al. has reported a similar finding stating that longer reaction time caused a growth on the c-axis of citrate-apatite particles resulting in long needle-like morphology [36]. Regardless of the trend, nanoparticles were obtained at two reaction times, at the second (Figure 4ii) and the eighth hour (Figure 4v). This complication on the nanostructure formation emerged from having both the crystallisation of calcium citrate and the absorption of **2a** occurring as well as the presence of ethanol. The experiment was verified again with the exact same reaction condition at 2, 5 and 8 h (Table 2). We suspect that the obtained nanoparticles at 2 h was the kinetic product that undergo phase change and reassembled into more thermodynamically favourable nanoparticles at 8 h. The phase change with respect to time is observed in calcium carbonate, where the amorphous calcium carbonate transforms into more stable vaterite then calcite over time [37]. The double nanoparticle formations could also be attributed to the presence of ethanol in the reaction. Ethanol was found to be able to stabilise vaterite phase of calcium carbonate preventing its rapid transformation to calcite. We suppose that this could be the case where the prior nanoparticles formation was maintained long enough to be observed by SEM [38]. The synthesised nanoparticles obtained from condition A and condition C had size consistent with those particles presented in Figure 4, but nanoparticles from condition A was slightly smaller than condition C at around 130 nm and 150 nm, respectively (Table 2). For condition B, the obtained particles were aggregated and plate-like with particle size over 1000 nm. Dynamic light scattering results also showed that the particles were monodispersed for both condition A and condition C, which the hydrodynamic diameter of nanoparticles were found to be in parallel to the SEM size (see Supplementary Materials). However, the values were larger due to the tendency of nanoparticles to agglomerate in aqueous solvent [39]. As the size of nanoparticles synthesised were between 300–370 nm, they still correspond to the desired size for nanoparticles to take advantages of the EPR effect.

**Figure 5:**
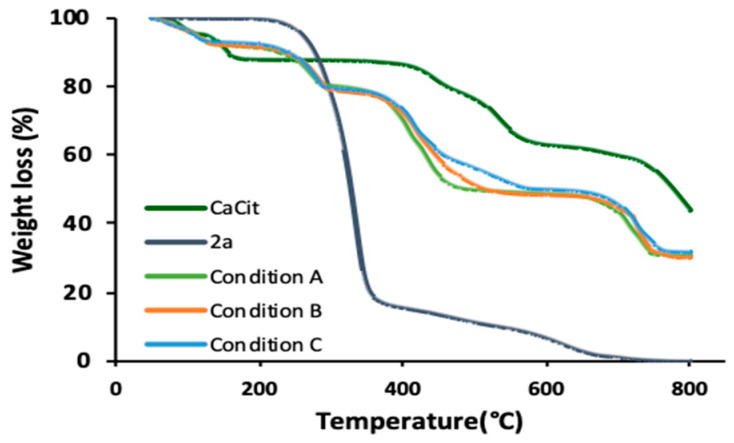
TGA curves of calcium citrate (CaCit), **2a** and Condition A–C.

The extent of drug incorporation in calcium citrate nanoparticles from condition A and condition C was calculated using CHNS elemental analysis. This method was chosen because **2a** was the only compound containing nitrogen, thus the percentage of nitrogen directly indicated the amount of **2a**. Condition B had the highest %drug loading at 17.8%, followed by condition C and condition A at 16.3% and 9.6%, respectively. It is noteworthy that despite the similarity of particle size for condition A and C, longer reaction time resulted in a higher %drug loading. Thermal gravimetric analysis additionally validated the success of drug incorporation. The TGA curves of calcium citrate nanoparticles, **2a** and particles obtained from condition A–C are illustrated in Figure 5. The decomposition of calcium citrate typically occurs in three stages. The first weight loss between 50–170 °C was caused by the release of exterior and interior water molecules [21,31]. The second stage between 420–600 °C indicated the change of calcium citrate to calcium carbonate. Lastly, the decomposition of calcium carbonate to calcium oxide occurred at temperature beyond 700 °C. For **2a**, it was almost a one-step decomposition starting at 200 °C. TGA curves of all obtained products (condition A–C) exhibited weight loss behaviours of both calcium citrate and **2a**. The weight loss between 50–150 °C attributed to release of moisture and the decrease in weight after 200 °C indicated the decomposition of **2a**. %weight loss for condition A and C agreed with the %drug loading calculated by CHNS analysis method at 13.5% and 15.6%, respectively. Overall, the optimised reaction condition to synthesise CaCit-triazine NPs preferred citric acid with NaOH as the source of citrate ion with reaction time of 8 h.

### 2.4. In Vitro Drug Release

**Figure 6:**
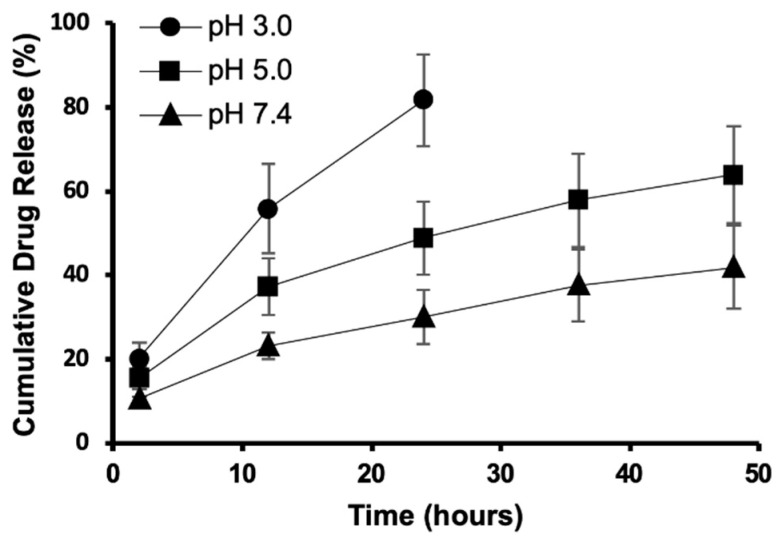
Release profile of 1,3,5-triazine incorporated calcium citrate nanoparticles (Condition C) at pH 3.0, 5.0 and 7.4.

The in vitro drug release study revealed that CaCit-**2a** NPs (condition C) was pH-responsive with controlled drug release behaviour over 48 h. The drug release performance was studied using dialysis method with 30%(*v*/*v*) EtOH in PBS as the release medium to accommodate the release of the hydrophobic 1,3,5-triazine derivative **2a** [40]. Typically, the physiological pH of blood and inside normal tissues is 7.4 and the pH of the tumour environment is slightly lower at 6.5–6.8 due to the metabolic adaptation called ‘Warburg effect’ where there is a higher rate of glycolytic metabolism producing an increasing number of protons (H^+^) [19]. Moreover, the lysosome of cancer cells has pH around 4.5 [41]. Therefore, we carried out the drug release at three conditions (pH = 7.4, 5.0, and 3.0) to study the effect of pH on the drug release. The general drug release trend illustrated that a higher %cumulative drug release was found at lower pH (Figure 6). The difference in drug release was attributed to the higher rate of calcium citrate decomposition in low pH condition [42]. After 24 h, 48.8% and 30.0% of **2a** was released for pH 5.0 and pH 7.4, respectively. A two-fold higher drug release was observed for pH 3.0 at 81.6%. The release of **2a** was sustained for 48 h with the release of over half of the nanoparticles’ loaded content at 63.9% for pH 5.0 and to a lesser extent of 41.9% for pH 7.4. It is probable that **2a** could still be released from CaCit-**2a** NPs even after 48 h.

## 3. Materials and Methods

### 3.1. Materials

Metformin hydrochloride and methyl cinnamate were purchased from Fluorochem (Hadfield, Derbyshire, UK); dicyandiamide, aniline, diethyl oxalate, methyl salicylate and sodium methoxide were purchased from TCI Chemical (Tokyo, Japan); isopropyl palmitate was purchased from Sigma Aldrich (St. Louis, MO, USA); methyl benzoate was gifted by the Department of Chemistry, Chulalongkorn University. Calcium chloride dihydrate and trisodium citrate dihydrate were purchased from Merck (Darmstadt, Germany). Citric acid anhydrous was purchased from Loba Chemie PVT. LTD (Mumbai, India). All of solvents used were distilled or analytical grade.

HCT116 (Human colorectal carcinoma) and SW620 (Human colorectal adenocarcinoma) cells were obtained from Nanomedicine RU, Faculty of Medicine, Chulalongkorn University. DMEM (Dulbecco’s Modified Eagle Medium), RPMI, fetal bovine serum (FBS), antibiotics, trypsin-EDTA, PBS (Phosphate Buffered Saline) (1X, pH 7.4) and Prestoblue™ cell viability reagent were purchased from Thermo Fisher Scientific (Waltham, MA, USA). Cisplatin was purchased from Sigma Aldrich (St. Louis, MO, USA) and dialysis bag Spectra/Por membranes (MWCO = 12–14 kDa) were purchased from Spectrum Laboratories, Inc. (Rancho Dominguez, CA, USA).

### 3.2. Methods

#### 3.2.1. Chemical Synthesis

The protocol for the synthesis of **2a**, **2b**, **2d**, **2f** and **3a** was modified from the one-pot reaction reported in the literature with slight adjustment of the ratio of the reagents and reaction time [10,15]. **2c**, **3b**, **3c**, **3d** and **3e** were synthesised using a modified two-step reaction with slight adjustment of the ratio of the reagents and reaction time [24,25].

*N^2^,N^2^-Dimethyl-6-pentadecyl-1,3,5-triazine-2,4-diamine* (**2a**): Metformin hydrochloride (497 mg, 3 mmol, 3 equiv.), NaOMe (1 mL, 5 mmol, 5 equiv.) and isopropyl palmitate (350 µL, 1 mmol, 1 equiv.) in anh. MeOH (4 mL) were mixed and heated under reflux for 2 h. Purification via extraction with EtOAc/H_2_O and silica gel column chromatography (eluent: 1:3 EtOAc:hexane) yielded **2a** as a white solid (225 mg, 64% yield). ^1^H-NMR (400 MHz, CDCl_3_) δ 5.17 (brs, 2H), 3.15 (brs, 3H), 3.11 (brs, 3H), 2.49 (t, *J* = 7.7 Hz, 3H), 1.78–1.64 (m, 2H), 1.32–1.19 (m, 24H), 0.87 (t, *J* = 6.7 Hz, 3H).; ^13^C-NMR (101 MHz, CDCl_3_) δ 178.1, 166.3, 165.6, 38.8, 36.3, 32.1, 29.8, 27.7 22.8, 14.2. Data are consistent with literature values [43].

*N^2^,N^2^-Dimethyl-6-phenyl-1,3,5-triazine-2,4-diamine* (**2b**): Metformin hydrochloride (497 mg, 3 mmol, 3 equiv.), NaOMe (1 mL, 5 mmol, 5 equiv.) and methyl benzoate (126 µL, 1 mmol, 1 equiv.) in anh. MeOH (4 mL) were heated under reflux for 2 h. The crude mixture purified by silica gel column chromatography (eluent: 1:2 EtOAc:hexane) yielded **2b** as a white-yellow solid. (112 mg, 52% yield). ^1^H-NMR (400 MHz, CDCl_3_) δ 8.37 (d, *J* = 7.5 Hz, 2H), 7.53–7.38 (m, 3H), 5.26 (brs, 2H), 3.30 (brs, 3H), 3.17 (brs, 3H); ^13^C-NMR (101 MHz, CDCl_3_) δ 171.0, 167.4, 166.0, 131.4, 130.1, 128.5, 128.3, 36.4. Data are consistent with literature values [7].

*2-(4-Amino-6-(dimethylamino)-1,3,5-triazin-2-yl)phenol* (**2c**): Metformin in the free base form was prepared with the same protocol as **1b** using metformin hydrochloride (3.313 g, 20 mmol, 1 equiv.) and NaOMe (4 mL, 20 mmol, 1 equiv.) in 15 mL of anh. MeOH. Next, excess amount of methyl salicylate (5 mL) was added and heated at 115 °C for 2 h with a notable formation of basic fume. Upon the completion of reaction, the crude mixture was acidified with NH_4_Cl to neutral pH then purified by extraction with EtOAc/H_2_O and silica gel column chromatography (eluent: 1:3, 1:2 EtOAc:hexane). The product was further purified by recrystallisation using EtOAc and hexane, which yielded **2c** as a greenish yellow solid. (2.153 g, 47% yield). ^1^H-NMR (400 MHz, CDCl_3_) δ 8.33 (d, *J* = 7.9 Hz, 1H), 7.37 (t, *J* = 7.8 Hz, 1H), 6.95 (d, *J* = 8.3 Hz, 1H), 6.89 (t, *J* = 7.5 Hz, 1H), 5.21 (brs, 2H), 3.22 (s, 3H), 3.17 (s, 3H); ^13^C-NMR (101 MHz, CDCl_3_) δ 171.3, 165.8, 164.4, 162.2, 134.1, 129.7, 119.1, 118.4, 118.1, 36.9, 36.7; IR (neat): 3390, 3324, 3200, 2926, 1652, 1566, 1499, 1031, 746; HRMS (ESI+): *m*/*z* calcd for C_11_H_13_N_5_O [M+H] ^+^ 232.1198, found 232.1191; Mp:194–197 °C.

*(E)-N^2^,N^2^-dimethyl-6-styryl-1,3,5-triazine-2,4-diamine* (**2d**): Metformin hydrochloride (331 mg, 2 mmol, 1 equiv.), NaOMe (800 µL, 4 mmol, 2 equiv.), and methyl cinnamate (446 µL, 3 mmol, 1.5 equiv.) in anh. MeOH (4 mL) were stirred under reflux for 3 h. The crude mixture purified by extraction using EtOAc/hexane yielded **2d** as a yellow solid (302 mg, quant.). ^1^H-NMR (400 MHz, CDCl_3_) δ 7.95 (d, *J* = 15.9 Hz, 1H), 7.58 (d, *J* = 7.0 Hz, 2H), 7.43–7.30 (m, 3H), 6.83 (d, *J* = 15.9 Hz, 1H), 5.28 (brs, 2H), 3.22 (brs, 3H), 3.15 (brs, 3H); ^13^C-NMR (101 MHz, CDCl_3_) δ 171.0, 167.2, 166.1, 139.7, 136.3, 129.6, 129.2, 128.3, 127.5, 36.7. Data are consistent with literature values [8].

*Methyl 4-amino-6-(dimethylamino)-1,3,5-triazine-2-carboxylate* (**2e**): Metformin in the free base form was prepared with the same protocol as **1b** using metformin hydrochloride (497 mg, 3 mmol, 1.5 equiv.) and NaOMe (400 µL, 2mmol, 1 equiv.) in 6 mL of anh. MeOH. Metformin dissolved in anh. MeOH (7.5 mL) was added dropwise into diethyl oxalate (1218 µL, 9 mmol, 3 equiv.) also dissolved in anh. MeOH (7.5 mL) with constant stirring motion at room temperature. Subsequently, the mixture was heated under reflux overnight. The mixture was purified by silica gel column chromatography (eluent: 3:5 EtOAc:hexane), which yielded **2e** as a yellow solid (27 mg, 7% yield). ^1^H-NMR (400 MHz, CDCl_3_) δ 5.68 (brs, 2H), 3.95 (s, 3H), 3.23 (s, 3H), 3.13 (s, 3H); ^13^C-NMR (101 MHz, CDCl_3_) δ 166.5, 165.4, 164.0, 163.1, 53.1, 36.4, 36.2; IR (neat): 3426, 3303, 3170, 2953, 1732, 1640, 1566, 1439, 1247, 1209, 1011, 793; HRMS (ESI+): *m*/*z* calcd for C_7_H_11_N_5_O_2_ [M+Na] ^+^ 220.0810, found 220.0815; Mp:219–221 °C.

*4-Amino-6-(dimethylamino)-1,3,5-triazine-2-carboxylic acid* (**2f**): Metformin hydrochloride (167 mg, 1 mmol, 1 equiv.), NaOMe (400 µL, 2 mmol, 2 equiv.) and diethyl oxalate (2975 µL, 22 mmol, 22 equiv.) in anh. MeOH (5 mL) were stirred under reflux for 4 h. The white precipitate was filtered, washed with MeOH and dried under vacuum to yield **2f** as a white-yellow solid (118 mg, 65% yield). ^1^H-NMR (400 MHz, DMSO) δ 11.08 (s, 1H), 9.01 (brs, 1H), 8.02 (brs, 1H), 3.11 (s, 3H), 3.02 (s, 3H); ^13^C-NMR (101 MHz, DMSO) δ 171.2, 169.9, 163.8, 160.4, 37.9, 37.2; IR (neat): 3304, 3168, 1763, 1708, 1563, 1479, 1324, 1040, 819; HRMS (ESI+): *m*/*z* calcd for C_6_H_9_N_5_O_2_ [M+Na] ^+^ 206.0654, found 206.0645; Mp: >300 °C.

*6-pentadecyl-N^2^-phenyl-1,3,5-triazine-2,4-diamine* (**3a**): **1a** (6.410 g, 30 mmol, 3 equiv.), NaOMe (10 mL, 50 mmol, 5 equiv.) and isopropyl palmitate (10.511 mL, 30 mmol, 3 equiv.) in anh. MeOH (40 mL) were stirred under reflux for 2 h. Purification via extraction with EtOAc/H_2_O and silica gel column chromatography (eluent: 1:3, 1:2 EtOAc:hexane) yielded **3a** as a white solid (5.0 g, 42% yield). ^1^H-NMR (400 MHz, DMSO) δ 9.38 (s, 1H), 7.77 (d, *J* = 7.9 Hz, 2H), 7.24 (t, *J* = 7.9 Hz, 2H), 6.94 (t, *J* = 7.2 Hz, 3H), 2.41 (t, *J* = 7.6 Hz, 2H), 1.73–1.60 (m, 2H), 1.29–1.20 (m, 23H), 0.84 (t, *J* = 6.7 Hz, 3H); ^13^C-NMR (101 MHz, DMSO) δ 177.8, 166.6, 164.2, 140.0, 128.3, 121.7, 119.7, 37.9, 31.2, 29.0, 26.9, 22.0, 13.9; IR (neat): 3462, 3311, 3106, 2913, 2848, 1655, 1626, 1528, 1470, 1429, 1385, 1028, 815, 747, 720, 699; HRMS (ESI+): *m*/*z* calcd for C_24_H_39_N_5_ [M+H] ^+^ 398.3284, found 398.3291; Mp: 118–121 °C.

*N^2^,6-diphenyl-1,3,5-triazine-2,4-diamine* (3b): **1b** was used without further purification. Methyl benzoate (792 µL, 6 mmol, 2 equiv.) was added into **1b** (532 mg, 3 mmol, 1 equiv.) dissolved in anh. MeOH (4 mL). The mixture was stirred under reflux for 24 h. Purified by silica gel column chromatography (eluent: 1:3 EtOAc:hexane) yielded **3b** as a yellow solid (54 mg, 7% yield). ^1^H-NMR (400 MHz, DMSO) δ 9.52 (s, 1H), 8.31 (d, *J* = 6.8 Hz, 2H), 7.84 (d, *J* = 8.0 Hz, 2H), 7.58–7.44 (m, 3H), 7.31 (t, *J* = 7.8 Hz, 2H), 7.12 (brs, 2H), 6.99 (t, *J* = 7.3 Hz, 1H); ^13^C-NMR (101 MHz, DMSO) δ 170.7, 167.6, 165.1, 140.3, 137.2, 131.8, 128.9, 128.7, 128.2, 122.5, 120.4. Data is consistent with literature values [44].

*2-(4-amino-6-(phenylamino)-1,3,5-triazin-2-yl)phenol* (**3c**): **1b** was used without further purification. A mixture of **1b** (355 mg, 2 mmol, 1 eqiv.) and an excess amount of methyl salicylate (500 µL) was stirred and heated at 115 °C for 7 h with a notable formation of basic fume. Upon the completion of reaction, the crude mixture was acidified with NH_4_Cl to neutral pH then purified by extraction with EtOAc/H_2_O and silica gel column chromatography (eluent: 1:4–1:2 EtOAc:hexane). The product was further purified by recrystallisation using EtOAc and hexane, which yielded **3c** as a greenish yellow solid (220 mg, 39% yield). ^1^H-NMR (400 MHz, DMSO) δ 13.49 (s, 1H), 9.77 (brs, 1H), 8.26 (d, *J* = 7.3 Hz, 1H), 7.76 (brs, 2H), 7.55 (brs, 1H), 7.44–7.35 (m, 2H), 7.33 (t, *J* = 7.2 Hz, 2H), 7.04 (t, *J* = 7.5 Hz, 1H), 6.90 (t, *J* = 8.0 Hz, 2H); ^13^C-NMR (101 MHz, DMSO) δ 169.9, 164.7, 160.8, 138.8, 133.2, 128.3, 128.1, 122.3, 120.2, 117.9, 117.2, 117.1; IR (neat): 3475, 3302, 3181, 1667, 1595, 1533, 1443 1429, 1031, 811, 752, 691; HRMS (ESI+): *m*/*z* calcd for C_15_H_13_N_5_O [M+H] ^+^ 280.1198, found 280.1205; Mp: 222–224 °C.

*(E)-N^2^-phenyl-6-styryl-1,3,5-triazine-2,4-diamine* (**3d**): **1b** was used without further purification. Methyl cinnamate (447 µL, 3 mmol, 1 equiv.) was added into **1b** (709 mg, 4 mmol, 1.33 equiv.) dissolved in anh. MeOH (4 mL). The mixture was stirred under reflux for 24 h. Purification by silica gel column chromatography (eluent: 1:2, 1:1 EtOAc:hexane) yielded **3d** as a yellow solid (154 mg, 17% yield). ^1^H-NMR (500 MHz) δ 9.47 (s, 1H), 7.87 (d, *J* = 15.9 Hz, 1H), 7.81 (dd, *J* = 8.7, 1.2 Hz, 2H), 7.66 (d, *J* = 7.5 Hz, 2H), 7.46–7.36 (m, 3H), 7.28 (t, *J* = 7.6 Hz, 2H), 7.03 (brs, 2H), 6.97 (tt, *J* = 7.3, 1.2 Hz, 1H), 6.82 (d, *J* = 16.0 Hz, 1H); ^13^C-NMR (101 MHz, CDCl_3_) δ 171.0, 166.6, 164.3, 141.2, 138.3, 135.5, 129.9, 129.1, 129.0, 128.2, 125.5, 124.0, 121.0; IR (neat): 3462, 3276, 3058, 1638, 1574, 1526, 1490, 1442, 970, 735, 691; HRMS (ESI+): *m*/*z* calcd for C_17_H_15_N_5_ [M+H] ^+^ 290.1406, found 290.1409; Mp: 186–189 °C.

*Methyl 4-amino-6-(phenylamino)-1,3,5-triazine-2-*carboxylate (**3e**): **1b** was used without further purification. **1b** (4.430 g, 25 mmol, 1 equiv.) dissolved in anh. MeOH (62.5 mL) was added dropwise into diethyl oxalate (10.15 mL, 75 mmol, 3 equiv.) also dissolved in anh. MeOH (62.5 mL) with constant stirring motion at room temperature. Subsequently, the mixture was stirred under reflux for 14 h. Purification by silica gel column chromatography (eluent: 1:1 EtOAc/hexane to 5% EtOAc/MeOH) yielded **3e** as a yellow solid (2.428 g, 40% yield). ^1^H-NMR (400 MHz, DMSO) δ 9.93 (brs, 1H), 7.78 (d, *J* = 7.9 Hz, 2H), 7.56 (brs, 1H), 7.43 (brs, 1H), 7.28 (t, *J* = 6.8 Hz, 2H), 7.01 (t, *J* = 7.9 Hz, 1H), 3.83 (s, 3H); ^13^C-NMR (101 MHz, DMSO) δ 167.3, 164.5, 164.2, 164.1, 139.5, 128.7, 122.7, 120.3, 52.6. Data are consistent with literature values [29].

*4-amino-6-(phenylamino)-1,3,5-triazine-2-carboxylic acid* (**3f**) was synthesised using a modified protocol from the literature [45]: NaOH (aq.) (50 mL, 1 M) was added to a solution of **3e** (2.023 g, 8.25 mmol) dissolved in EtOH (100 mL). The mixture was stirred under reflux for 3 h, cooled to room temperature, followed by the addition of HCl (aq.) (50 mL, 1M). The resulting precipitate was filtered, washed with DI and dried under vacuum to yield **3f** as a pale-yellow solid (2.416 g, quant.). ^1^H-NMR (400 MHz, DMSO) δ 9.89 (brs, 1H), 7.78 (d, *J* = 8.1 Hz, 2H), 7.47 (brs, 2H), 7.28 (t, *J* = 7.3 Hz, 2H), 7.00 (t, *J* = 7.5, 6.7 Hz, 1H); ^13^C-NMR (101 MHz, DMSO) δ 166.9, 165.4, 165.0, 164.3, 139.4, 128.5, 122.6, 120.2; IR (neat): 3319, 3129, 1675, 1622, 1594, 1568, 1489, 1451, 1005, 788, 761, 691; HRMS (ESI+): *m*/*z* calcd for C_10_H_9_N_5_O_2_ [M+2Na-H] ^+^ 276.0473, found 276.0473; Mp: 248–249 °C.

#### 3.2.2. Characterisation of 1,3,5-Triazine Derivatives

^1^H and ^13^C-NMR were recorded with Bruker Avance (III) 400WB spectrometer (400 mHz) and JEOL JNM-ECZS NMR spectrometer (500 mHz); FTIR spectra were analysed by Thermo Scientific™ Nicolet 6700 FT-IR Spectrometer with ATR mode (32 scans); high-resolution mass spectra (HRMS) were recorded with micrOTOF-Q II mass spectrometer (Bruker Daltonics) with electrospray ionization; and melting points (Mp) were measured using a Stuart SMP20 melting point apparatus.

#### 3.2.3. In vitro Anticancer Activity of 1,3,5-Triazine Derivatives

HCT116 were cultured in a growth medium of RPMI. SW620 were cultured in a growth medium of DMEM. Both cell culture mediums were supplemented with 10% FBS and 1% antibiotic, and the cells were maintained in an incubator at 37 °C in a humidified atmosphere of 5% CO_2_.

HCT116 and SW620 were seeded in 96-well plates at a density of 1 × 10^4^ per well in cell culture medium and incubated for 24 h to allow cell adherence. Cells were treated with the synthesised 1,3,5-triazine derivatives at concentration between 0–100 µM for 48 h. Following incubation, 10 µL PrestoBlue™ solution was added to each well, and then plates were placed back into the incubator for a further 30 min incubation [46]. Fluorescence was measured using a microplate reader at 560 nm excitation and 590 nm emission (Thermo, Varioskan Flash, England). The results were analysed by one-way ANOVA and Tukey post-test using GraphPad Prism 5.0 (Graph-Pad Software Inc., San Diego, CA, USA).

#### 3.2.4. Preparation of 1,3,5-Triazine Incorporated Calcium Citrate Nanoparticles (Condition A–C)

Solution A: citric acid was first dissolved in DI water, followed by the addition of 1M NaOH until pH of solution reached pH 6.0 then the volume was adjusted to 5 mL with EtOH.; Solution B: **2a** was dissolved in 5 mL EtOH while heating at 50–60 °C.; Solution C: calcium chloride was dissolved in 5 mL EtOH. Solution A was added into solution B which turned cloudy straightaway. EtOH and DI water were added to the mixture to obtain a clear solution. Solution C was added into the AB mixture with vigorous stirring then moved to stir at room temperature for 2, 5 or 8 h. The white suspension was centrifuged, washed with EtOH/DI water and freeze dried for 24 h.

#### 3.2.5. Characterisation of 1,3,5-Triazine Incorporated Calcium Citrate Nanoparticles

Size and morphology of the synthesised products were recorded with JEOL JSM-6480LV scanning electron microscope at 15kV; hydrodynamic size and zeta potential were measured with the Zetasizer Nano ZS, Malvern Instrument (IR of 1.660); functional groups were analysed by Thermo Scientific™ Nicolet 6700 FT-IR Spectrometer (Thermo Fisher Scientific, Waltham, MA, USA) with ATR mode; chemical changes or decomposition temperature by Perkin Elmer Pyris™ 1 TGA; elemental analysis by Thermo Flash 2000 CHNS/O analyser; and %cumulative drug release was measured with UV/Vis spectrometer (Hewlett Packard 8453, Agilent Technology, Santa Clara, CA, USA).

#### 3.2.6. Drug Release Study

The drug release profile of the synthesised nanoparticles was determined by the dialysis method carried out at 37 °C and stirring speed of 100 rpm. Thirty percent (*v*/*v*) of EtOH in PBS at pH 3.0, 5.0 and 7.4 were used as the release medium [40]. Nanoparticles (weight equivalent to 1 mg of **2a**) were dispersed in 10 mL 30% (*v*/*v*) EtOH/PBS at pH 3.0, 5.0 and 7.4. The dialysis bag was immersed into 190 mL of release medium. At predetermined time, the dialysis bag was moved into another container with 190 mL of fresh release medium [22]. To extract **2a** out of the release medium, the solution was evaporated under vacuum to remove EtOH and extracted with dichloromethane [47]. For the UV-Vis spectroscopy measurement, 10 mL of 60% EtOH/PBS acidified with HCl to pH 3.0, 5.0 and 7.4 was added to each sample and measured at 200–320 nm. The calibration curve was drawn using a standard which was **2a** dissolved in 60% EtOH/PBS at various concentrations.

## 4. Conclusions

In conclusion, we have reported a new study that integrated drug discovery with drug delivery. Twelve 1,3,5-triazine derivatives of metformin (**2a**–**2f**) and phenylbiguanide (**3a**–**3f**) were synthesised via two pathways using various esters. The anticancer activity of the derivatives ranged widely depending on the hydrophobicity of the substituent. It was found that some of the derivatives were more potent as anticancer agents with IC_50_ comparable to the values of the reference drug, cisplatin. The optimisation for the preparation of CaCit-**2a** NPs under EtOH-water binary solvent system revealed that the use of 1:2 citric acid/NaOH as the citrate source with a reaction time of 8 h provided the best result. The obtained nanoparticles had appropriate size satisfying the EPR effect, moderate %drug loading, as well as showing a controlled drug release behaviour under pH 5.0 environment. The knowledge from both the biological evaluations and the DDS studies could be valuable for further development of triazine-based anticancer drug and could serve as a platform for potential calcium citrate nanoparticles drug delivery system in the future.

## Figures and Tables

**Table 1:** Anticancer activities of synthesised 1,3,5-triazine derivatives.

1,3,5-Triazine Derivatives	%Cytotoxicity at 100 µM ^1^		IC_50_ (µM) ^2^	
	SW620	HCT116	SW620	HCT116
**1a**	n.a. ^3^	n.a. ^3^	n.d. ^4^	n.d. ^4^
**2a**	96.02 ± 0.06	67.43 ± 1.82	59.13 ± 1.46	59.35 ± 1.66
**2b**	15.86 ± 1.91	19.23 ± 5.94	n.d.	n.d.
**2c**	53.85 ± 1.40	50.77 ± 1.83	25.25 ± 1.12	26.29 ± 1.07
**2d**	27.39 ± 4.16	26.52 ± 3.77	n.d. ^4^	n.d. ^4^
**2e**	11.41 ± 3.52	7.16 ± 2.11	n.d. ^4^	n.d. ^4^
**2f**	5.64 ± 1.51	11.59 ± 4.03	n.d. ^4^	n.d. ^4^
**3a**	54.96 ± 1.46	66.56 ± 2.58	54.55 ± 1.09	59.17 ± 1.48
**3b**	52.60 ± 4.06	54.42 ± 8.48	38.37 ± 1.05	26.92 ± 1.31
**3c**	56.85 ± 3.18	65.46 ± 1.07	22.80 ± 1.06	20.79 ± 1.07
**3d**	87.47 ± 1.54	92.27 ± 0.04	54.10 ± 1.05	55.02 ± 1.04
**3e**	12.14 ± 3.40	5.54 ± 3.65	n.d. ^4^	n.d. ^4^
**3f**	15.36 ± 1.15	14.22 ± 2.81	n.d. ^4^	n.d. ^4^
**Cisplatin**	62.14 ± 5.05	78.20 ± 0.96	31.67 ± 1.13	19.18 ± 1.06

^1^ Results expressed in percentage taken as a mean of triplicates ± standard deviation (SD). ^2^ Results expressed in µmol/L (µM), taken as a mean value of triplicates ± standard deviation (SD). ^3^ Not active at the specified. ^4^ Not determined.

**Table 2:** Characterisation of 1,3,5-triazine incorporated calcium citrate nanoparticles.

Condition	Reaction Time (h)	SEM Size (nm)	Particle Size (nm) [PDI]	Zeta Potential (mV)	Drug Loading (%)	
A	2	134 ± 18.87	369.30 ± 13.44 [0.464]	−8.36	9.6 ^1^	13.5 ^2^
B	5	>1000	n.d. ^3^	n.d. ^3^	17.8 ^1^	n.d. ^3^
C	8	148 ± 23.69	300.30 ± 12.42 [0.322]	−7.71	16.3 ^1^	15.6 ^2^
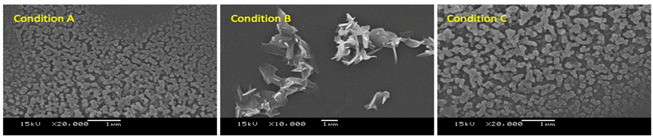						

^1^ %drug loading calculated by CHNS elemental analysis. ^2^ %drug loading calculated by thermal gravimetric analysis. ^3^ Not determined.

## Data Availability

Data is contained within the article or Supplementary Material.

## Supplementary Materials

The following are available online, ^1^H and ^13^C-NMR spectra for compounds **1a**, **1b**, **2a**–**2f** and **3a**–**3f**; IR and HRMS spectra for compound **2c**, **2e**, **2f**, **3a**, **3c**, **3d**, and **3f**; biological evaluations of **2a**–**2f** and **3a**–**3f**; size distribution graphs for Condition A and C.

## Sample Availability

Samples of the compounds **2a**, **2c**, and **3c** are available from the authors.

## References

[1] Siegel R.L., Miller K.D., Jemal A. (2019). Cancer statistics, 2019. Ca: Cancerj. Clin..

[2] Das M., Mohanty C., Sahoo S.K. (2009). Ligand-based targeted therapy for cancer tissue. Expert Opin. Drug Deliv..

[3] Banerjee A., Pathak S., Subramanium V.D., Dharanivasan G., Murugesan R., Verma R.S. (2017). Strategies for targeted drug delivery in treatment of colon cancer: Current trends and future perspectives. Drug Discov. Today.

[4] Singla P., Luxami V., Paul K. (2016). Synthesis and in vitro evaluation of novel triazine analogues as anticancer agents and their interaction studies with bovine serum albumin. Eur. J. Med. Chem..

[5] Junaid A., Lim F.P.L., Tiekink E.R.T., Dolzhenko A.V. (2020). Design, synthesis, and biological evaluation of new 6,N_2_-diaryl-1,3,5-triazine-2,4-diamines as anticancer agents selectively targeting triple negative breast cancer cells. Rsc Adv..

[6] Junaid A., Lim F.P.L., Tiekink E.R.T., Dolzhenko A.V. (2019). New One-Pot Synthesis of 1,3,5-Triazines: Three-Component Condensation, Dimroth Rearrangement, and Dehydrogenative Aromatization. Acs Comb. Sci..

[7] Zeng M., Wang T., Cui D.-M., Zhang C. (2016). Ruthenium-catalyzed synthesis of tri-substituted 1,3,5-triazines from alcohols and biguanides. Newj. Chem..

[8] Zeng M., Xie Z.P., Cui D.-M., Zhang C. (2018). Ruthenium-catalyzed synthesis of arylethyl 1,3,5-triazines from arylallyl alcohols and biguanides. Org. Biomol. Chem..

[9] Xu Y., Shen B., Liu L., Qiao C. (2020). Metal free [4+1] and [5+1] annulation reactions to prepare heterocycles using DMF and its derivatives as one-carbon source. Tetrahedron Lett..

[10] Cao H., Liao S., Zhong W., Xiao X., Zhu J., Li W., Wu X., Feng Y. (2017). Synthesis, Characterization, and Biological Evaluations of 1,3,5-Triazine Derivatives of Metformin Cyclization with Berberine and Magnolol in the Presence of Sodium Methylate. Molecules.

[11] Makowska A., Sączewski F., Bednarski P.J., Sączewski J., Balewski Ł. (2018). Hybrid Molecules Composed of 2,4-Diamino-1,3,5-triazines and 2-Imino-Coumarins and Coumarins. Synthesis and Cytotoxic Properties. Molecules.

[12] Chaurasia S.R., Dange R., Bhanage B.M. (2020). Graphene oxide as a carbo-catalyst for the synthesis of tri-substituted 1,3,5-triazines using biguanides and alcohols. Catal. Commun..

[13] Yao W., Duan Z.-C., Zhang Y., Sang X., Xia X.-F., Wang D. (2019). Iridium Supported on Phosphorus-Doped Porous Organic Polymers: Active and Recyclable Catalyst for Acceptorless Dehydrogenation and Borrowing Hydrogen Reaction. Adv. Synth. Catal..

[14] Koh M., Lee J.-C., Min C., Moon A. (2013). A novel metformin derivative, HL010183, inhibits proliferation and invasion of triple-negative breast cancer cells. Bioorg. Med. Chem..

[15] Kothayer H., Spencer S.M., Tripathi K., Westwell A.D., Palle K. (2016). Synthesis and in vitro anticancer evaluation of some 4,6-diamino-1,3,5-triazine-2-carbohydrazides as Rad6 ubiquitin conjugating enzyme inhibitors. Bioorg. Med. Chem. Lett..

[16] Torchilin V. (2011). Tumor delivery of macromolecular drugs based on the EPR effect. Adv. Drug Deliv. Rev..

[17] Hickey J.W., Santos J.L., Williford J.-M., Mao H.-Q. (2015). Control of polymeric nanoparticle size to improve therapeutic delivery. J. Control. Release.

[18] Bae Y.H., Park K. (2011). Targeted drug delivery to tumors: Myths, reality and possibility. J. Control. Release.

[19] Estrella V., Chen T., Lloyd M., Wojtkowiak J., Cornnell H.H., Ibrahim-Hashim A., Bailey K., Balagurunathan Y., Rothberg J.M., Sloane B.F. (2013). Acidity Generated by the Tumor Microenvironment Drives Local Invasion. Cancer Res..

[20] Zhang W., Wang W., Chen Q.-Y., Lin Z.-Q., Cheng S.-W., Kou D.-Q., Ying X.-Z., Shen Y., Cheng X.-J., Nie P.-F. (2012). Effect of calcium citrate on bone integration in a rabbit femur defect model. Asian Pac. J. Trop. Med..

[21] Li J., Liu Y., Gao Y., Zhong L., Zou Q., Lai X. (2016). Preparation and properties of calcium citrate nanosheets for bone graft substitute. Bioengineered.

[22] Oungeun P., Rojanathanes R., Pinsornsak P., Wanichwecharungruang S. (2019). Sustaining Antibiotic Release from a Poly(methyl methacrylate) Bone-Spacer. Acs Omega.

[23] Rimsueb N., Cherdchom S., Aksornkitti V., Khotavivattana T., Sereemaspun A., Rojanathanes R. (2020). Feeding Cells with a Novel “Trojan” Carrier: Citrate Nanoparticles. Acs Omega.

[24] Kothayer H., Morelli M., Brahemi G., Elshanawani A.A., Abu Kull M.E., El-Sabbagh O.I., Shekhar M.P.V., Westwell A.D. (2014). Optimised synthesis of diamino-triazinylmethyl benzoates as inhibitors of Rad6B ubiquitin conjugating enzyme. Tetrahedron Lett..

[25] Shapiro S.L., Parrino V.A., Freedman L. (1961). Guanamines. VIII. 6-(Substituted Phenyl)guanamines. J. Org. Chem..

[26] Mallon R., Feldberg L.R., Lucas J., Chaudhary I., Dehnhardt C., Santos E.D., Chen Z., dos Santos O., Ayral-Kaloustian S., Venkatesan A. (2011). Antitumor Efficacy of PKI-587, a Highly Potent Dual PI3K/mTOR Kinase Inhibitor. Clin. Cancer Res..

[27] Moreno L.M., Quiroga J., Abonia R., Ramírez-Prada J., Insuasty B. (2018). Synthesis of New 1,3,5-Triazine-Based 2-Pyrazolines as Potential Anticancer Agents. Molecules.

[28] Zhou J., Li P., Xue X., He S., Kuang Y., Zhao H., Chen S., Zhi Q., Guo X. (2013). Salinomycin induces apoptosis in cisplatin-resistant colorectal cancer cells by accumulation of reactive oxygen species. Toxicol. Lett..

[29] Kothayer H., Elshanawani A.A., Abu Kull M.E., El-Sabbagh O.I., Shekhar M.P.V., Brancale A., Jones A.T., Westwell A.D. (2013). Design, synthesis and in vitro anticancer evaluation of 4,6-diamino-1,3,5-triazine-2-carbohydrazides and -carboxamides. Bioorg. Med. Chem. Lett..

[30] Peng H., Li K., Wang T., Wang J., Wang J., Zhu R., Sun D., Wang S. (2013). Preparation of hierarchical mesoporous CaCO3 by a facile binary solvent approach as anticancer drug carrier for etoposide. Nanoscale Res. Lett..

[31] Li J.F., Gao Y., Zhong L.Z., Liu Y.Q., Liu H.Q., Zou Q., Lai X.F. (2017). Facile Self-Assembly Synthesis of Hierarchical 3D Flowerlike Calcium Citrate Microspheres. J. Nano Res..

[32] Garcia A.C., Vavrusova M., Skibsted L.H. (2018). Supersaturation of calcium citrate as a mechanism behind enhanced availability of calcium phosphates by presence of citrate. Food Res. Int..

[33] Um N., Hirato T. (2014). Precipitation behavior of Ca(OH)_2_, Mg(OH)_2_, and Mn(OH)_2_ from CaCl_2_, MgCl_2_, and MnCl_2_ in NaOH-H_2_O solutions and study of lithium recovery from seawater via two-stage precipitation process. Hydrometallurgy.

[34] Al-Khaldi M.H., Nasr-El-Din H.A., Mehta S., Al-Aamri A.D. (2007). Reaction of citric acid with calcite. Chem. Eng. Sci..

[35] Ma C., Gerhard E., Lu D., Yang J. (2018). Citrate chemistry and biology for biomaterials design. Biomaterials.

[36] Iafisco M., Ramírez-Rodríguez G.B., Sakhno Y., Tampieri A., Martra G., Gómez-Morales J., Delgado-López J.M. (2015). The growth mechanism of apatite nanocrystals assisted by citrate: Relevance to bone biomineralization. CrystEngComm.

[37] Xiao H., Hu C., Chen C., Tao C., Wu Y., Jiang J. (2020). The advantage of alcohol–calcium method on the formation and the stability of vaterite against ethanol–water binary solvent method. J. Mater. Res..

[38] Sand K.K., Rodriguez-Blanco J.D., Makovicky E., Benning L.G., Stipp S.L.S. (2012). Crystallization of CaCO_3_ in Water–Alcohol Mixtures: Spherulitic Growth, Polymorph Stabilization, and Morphology Change. Cryst. Growth Des..

[39] Hebeish A., El-Rafie M.H., El-Sheikh M.A., El-Naggar M.E. (2014). Ultra-Fine Characteristics of Starch Nanoparticles Prepared Using Native Starch With and Without Surfactant. J. Inorg Organomet Polym Mater..

[40] Hardiansyah A., Yang M.-C., Liu T.-Y., Kuo C.-Y., Huang L.-Y., Chan T.-Y. (2017). Hydrophobic Drug-Loaded PEGylated Magnetic Liposomes for Drug-Controlled Release. Nanoscale Res. Lett..

[41] Zhou C., Chen T., Wu C., Zhu G., Qiu L., Cui C., Hou W., Tan W. (2015). Aptamer CaCO_3_ Nanostructures: A Facile, pH-Responsive, Specific Platform for Targeted Anticancer Theranostics. Chem. Asianj..

[42] Guo Y., Li H., Shi W., Zhang J., Feng J., Yang X., Wang K., Zhang H., Yang L. (2017). Targeted delivery and pH-responsive release of doxorubicin to cancer cells using calcium carbonate/hyaluronate/glutamate mesoporous hollow spheres. J. Colloid Interface Sci..

[43] Liu C. (2005). Synthesis of the derivatives of 2-Amino-4-dimethylamino-1,3,5-triazine. J. Guangdong Coll. Pharm..

[44] Liu C., Lin J., Leftheris K. (2007). A novel one-pot synthesis of N,6-disubstituted 1,3,5-triazine-4,6-diamines from isothiocyanates and amidines. Tetrahedron Lett..

[45] Lebel O., Perron M.-È., Maris T., Zalzal S.F., Nanci A., Wuest J.D. (2006). A New Class of Selective Low-Molecular-Weight Gelators Based on Salts of Diaminotriazinecarboxylic Acids. Chem. Mater..

[46] Xu M., McCanna D.J., Sivak J.G. (2015). Use of the viability reagent PrestoBlue in comparison with alamarBlue and MTT to assess the viability of human corneal epithelial cells. J. Pharmacol. Toxicol. Methods.

[47] Wu J.-L., Wang C.-Q., Zhuo R.-X., Cheng S.-X. (2014). Multi-drug delivery system based on alginate/calcium carbonate hybrid nanoparticles for combination chemotherapy. Colloids Surf. B.

