# Supplementary material for: Biguanide-Based Synthesis of 1,3,5-Triazine Derivatives with Anticancer Activity and 1,3,5-Triazine Incorporated Calcium Citrate Nanoparticles
Source: Molecules. 2021 Feb 15;26(4):1028. doi: 10.3390/molecules26041028 (PMC7919653; doi:10.3390/molecules26041028)
Supplement: Supplementary file 1 [file molecules-26-01028-s001.pdf]

Supplementary Information

Biguanide-based synthesis of 1,3,5-triazine derivatives with anticancer activity and 1,3,5-triazine incorporated calcium citrate nanoparticles

Monnaya Chalermnon,[a] Sarocha Cherdchom,[b,c] Amornpun Sereemaspun,[d] Rojrit Rojanathanes,[e] and Tanatorn Khotavivattana*[f]

a. Faculty of Science, Department of Chemistry, Chulalongkorn University, Bangkok 10330, Thailand

b. Department of Pharmacy Practice, Faculty of Pharmaceutical Sciences, Chulalongkorn University, Phayathai road, Wangmai, Patumwan, Bangkok 10330, Thailand

c. NanoMedicine Research Unit, Department of Anatomy, Faculty of Medicine, Chulalongkorn University, Rama 4 Road, Patumwan, Bangkok 10330, Thailand

d. Chula Medical Innovation Centre (CMIC), Nanomedicine Research Unit, Department of Anatomy, Faculty of Medicine, Chulalongkorn University, Bangkok 10330, Thailand

e. Centre of Excellence in Materials and Bio-Interfaces Faculty of Science, Chulalongkorn University, Bangkok 10330, Thailand

f. Centre of Excellence in Natural Products Chemistry, Department of Chemistry, Chulalongkorn University, Bangkok 10330, Thailand

**Co-corresponding authors*

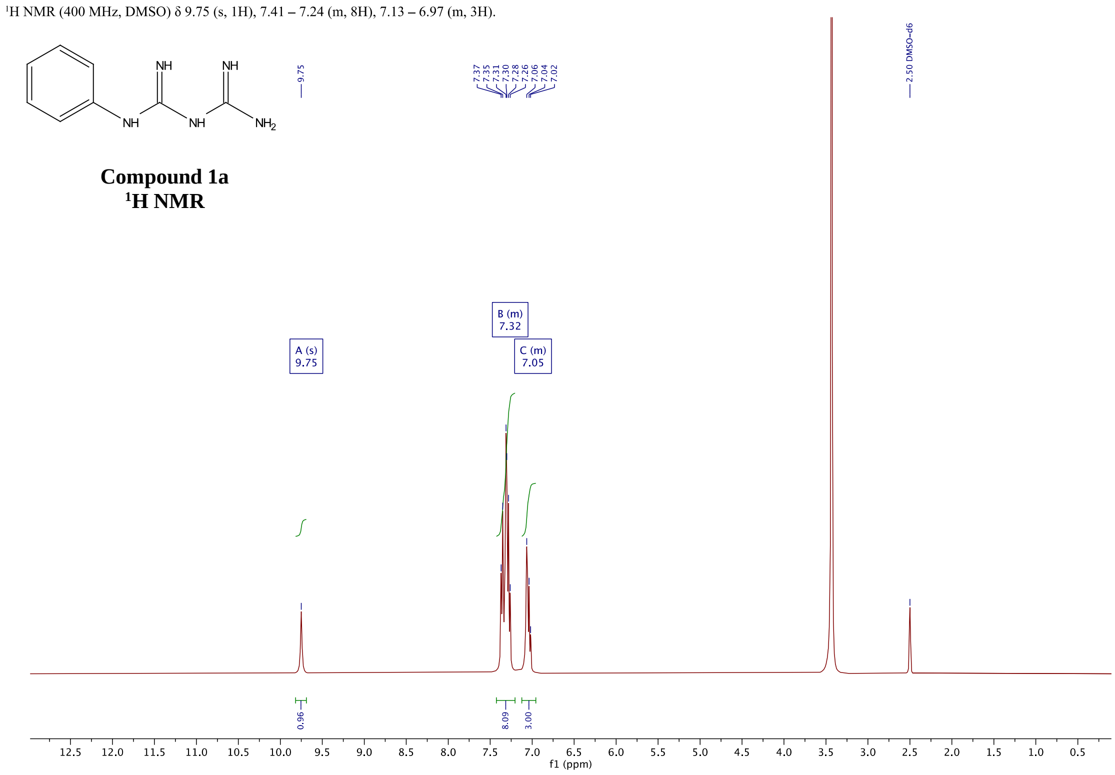

Figure 1. ^{1}H NMR of Phenylbiguanide hydrochloride (**1a**)

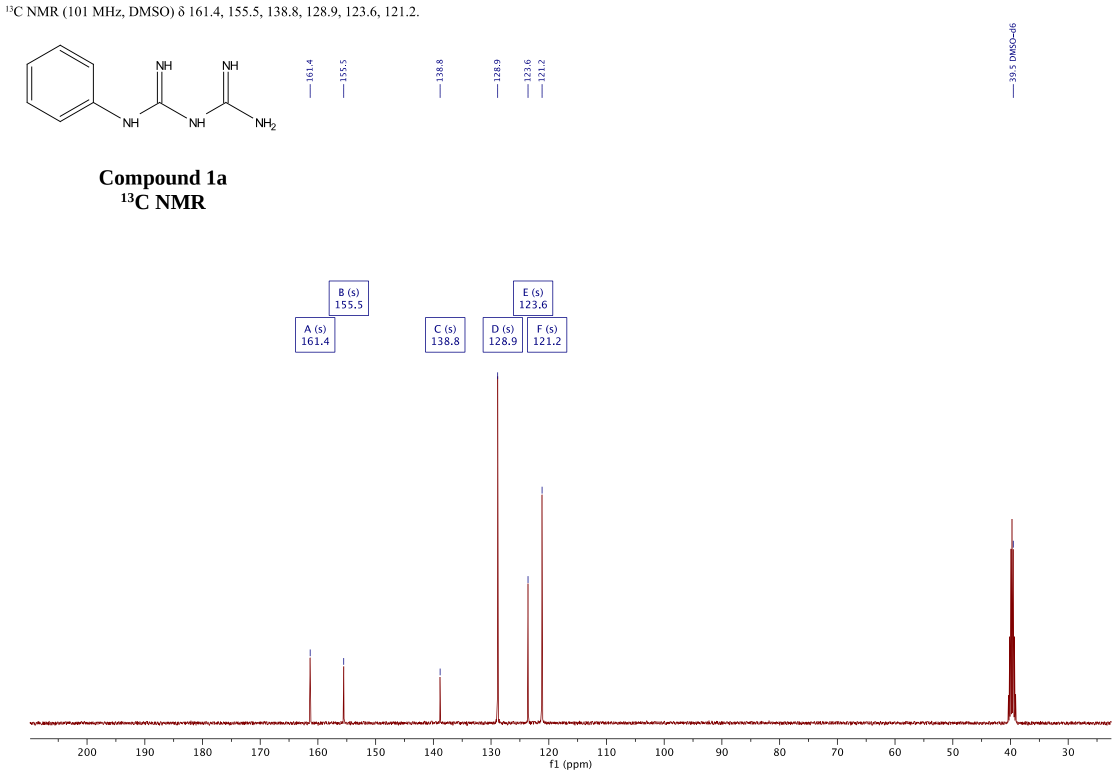

Figure 2. ^{13}C NMR of Phenylbiguanide hydrochloride (**1a**)

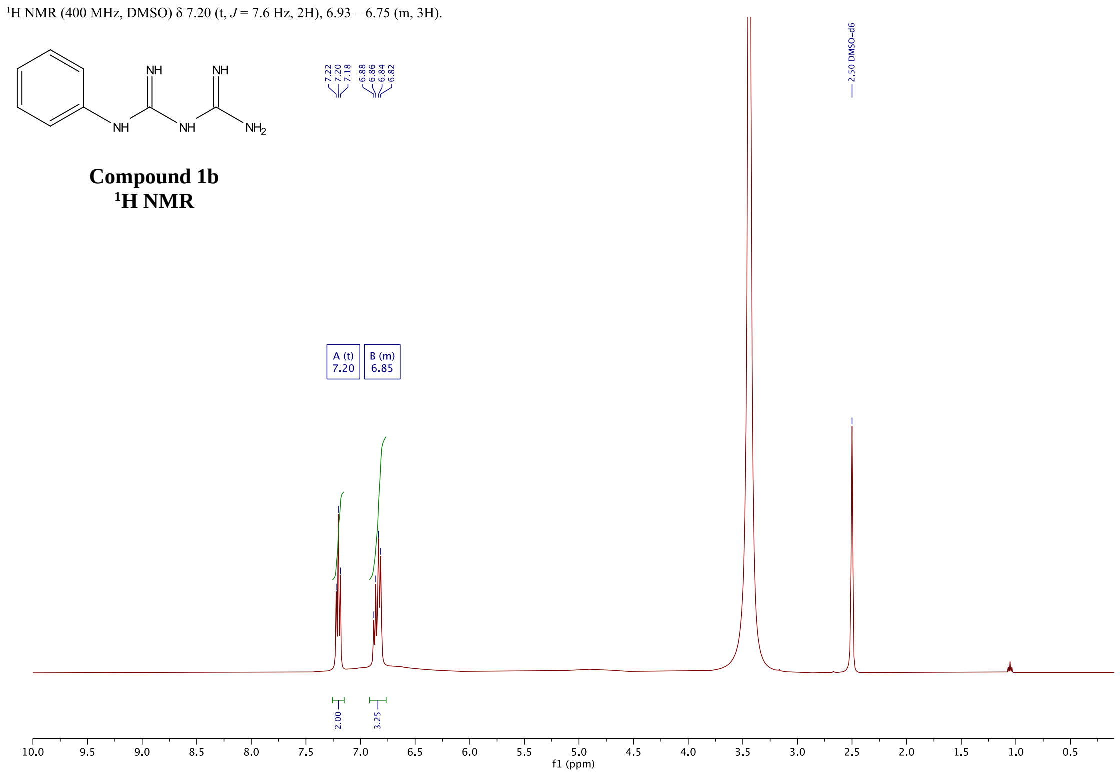

Figure 3. ^{1}H NMR of Phenylbiguanide (**1b**)

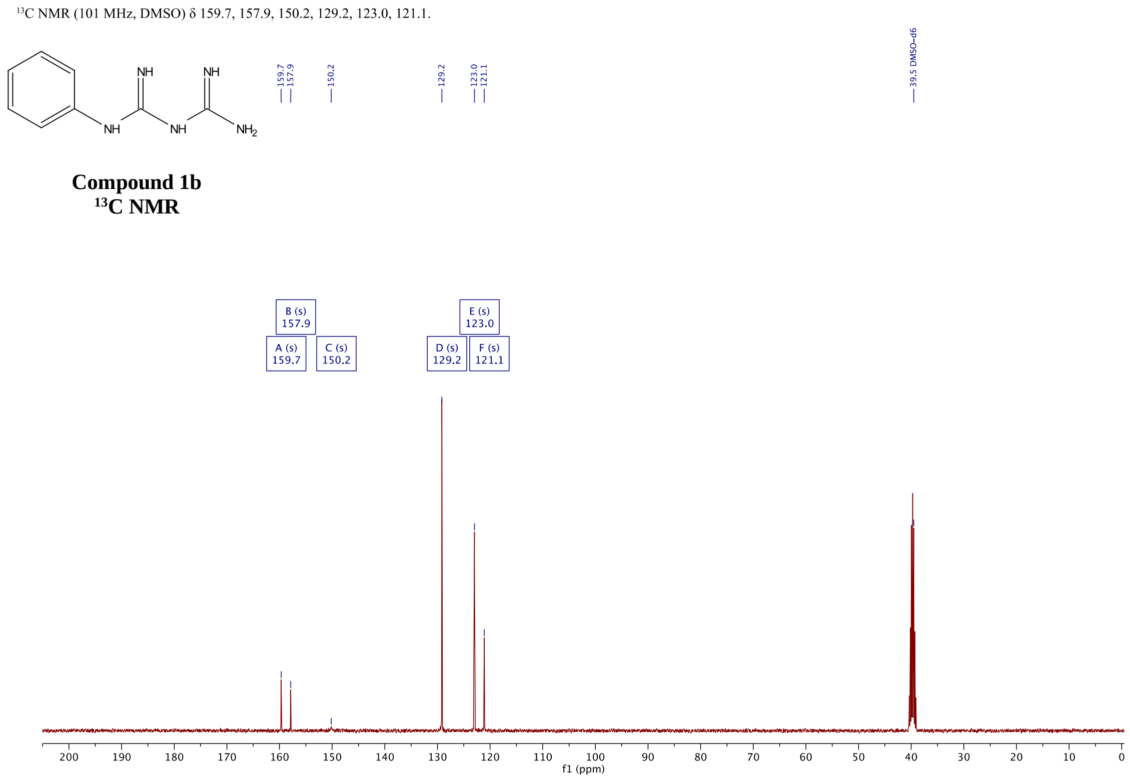

Figure 4. ^{13}C NMR of Phenylbiguanide (**1b**)

^{1}H NMR (400 MHz, $CDCl_3$) δ 5.17 (brs, 2H), 3.15 (brs, 3H), 3.11 (brs, 3H), 2.49 (t, *J* = 7.7 Hz, 3H), 1.78 – 1.64 (m, 2H), 1.32 – 1.19 (m, 24H), 0.87 (t, *J* = 6.7 Hz, 3H).

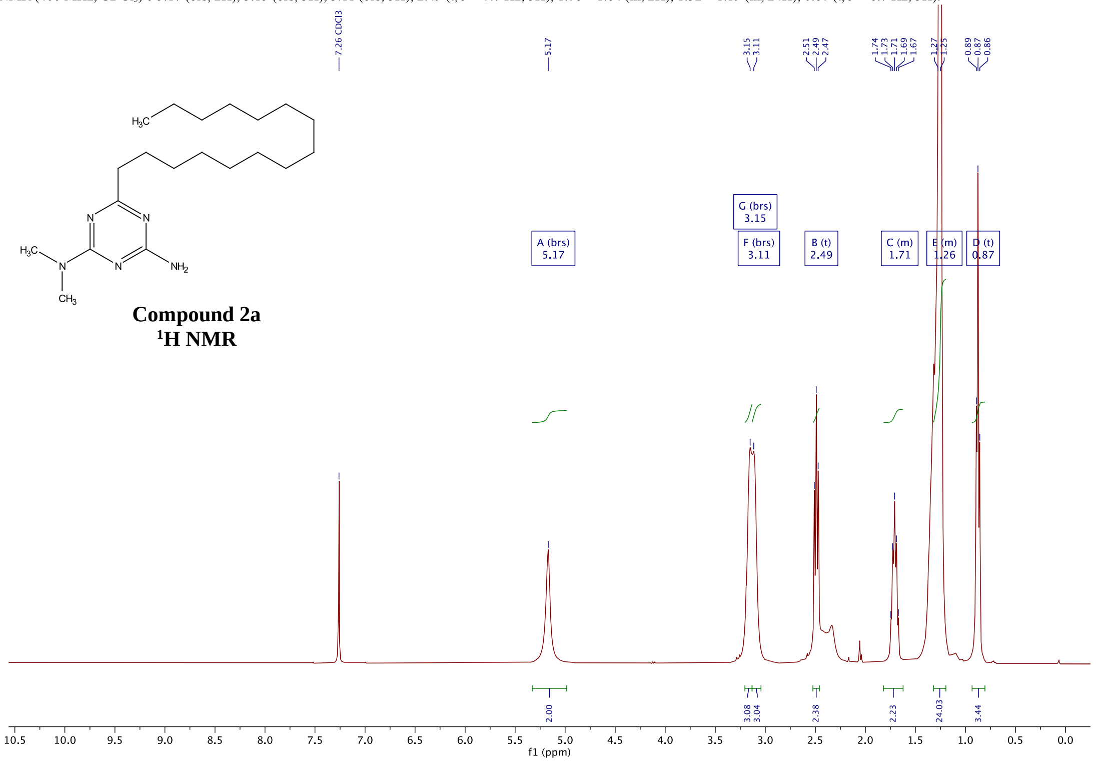

Figure 5. ^{1}H NMR of N^2,N^2-dimethyl-6-pentadecyl-1,3,5-triazine-2,4-diamine (**2a**)

^{13}C NMR (101 MHz, $CDCl_3$) δ 178.1, 166.3, 165.6, 38.8, 36.3, 32.1, 29.8, 27.7 22.8, 14.2.

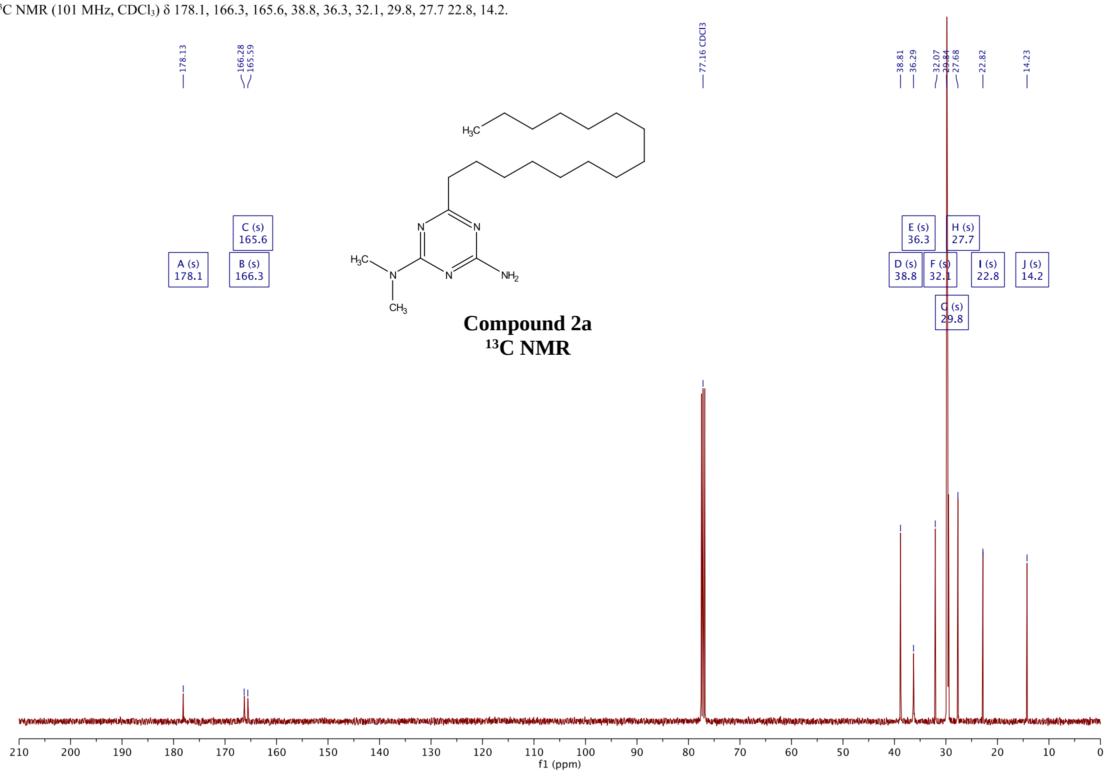

Figure 6. ^{13}C NMR of N^2,N^2-dimethyl-6-pentadecyl-1,3,5-triazine-2,4-diamine (**2a**)

^{1}H NMR (400 MHz, $CDCl_3$) δ 8.37 (d, *J* = 7.5 Hz, 2H), 7.53 – 7.38 (m, 3H), 5.26 (brs, 2H), 3.30 (brs, 3H), 3.17 (brs, 3H).

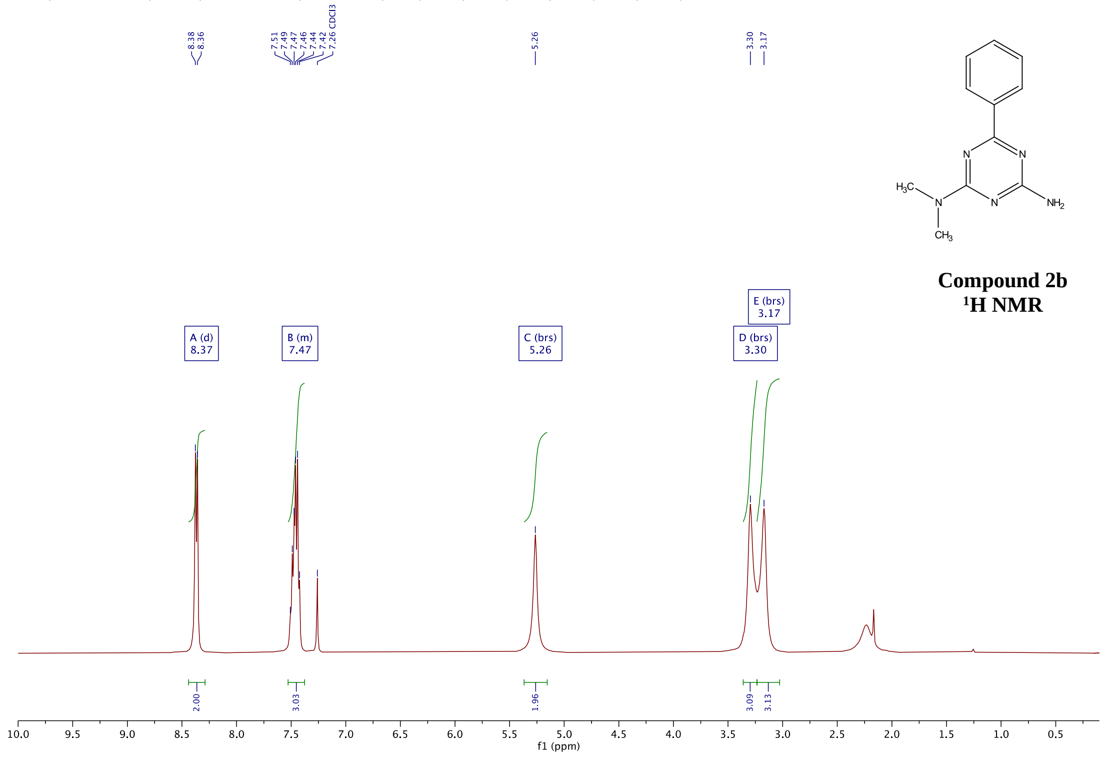

Figure 7. ^{1}H NMR of N^2,N^2-dimethyl-6-phenyl-1,3,5-triazine-2,4-diamine (**2b**)

^{13}C NMR (101 MHz, $CDCl_3$) δ 171.0, 167.4, 166.0, 131.4, 130.1, 128.5, 128.3, 36.4.

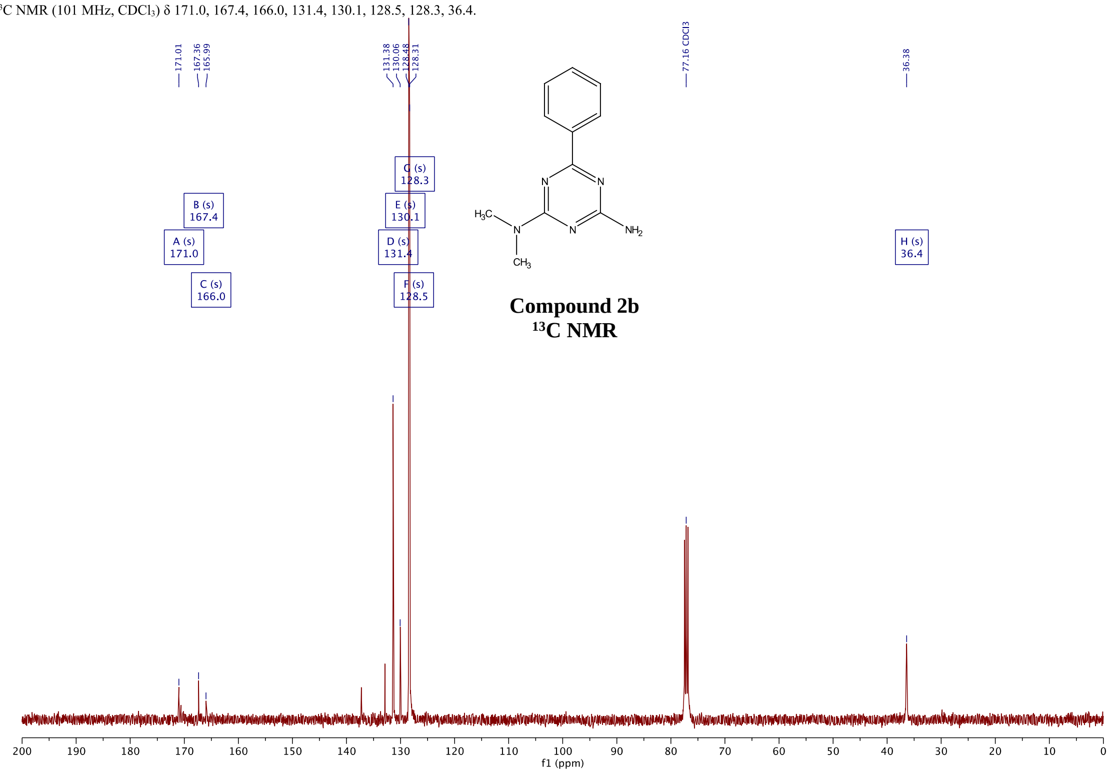

Figure 8. ^{13}C NMR of N^2,N^2-dimethyl-6-phenyl-1,3,5-triazine-2,4-diamine (**2b**)

^{1}H NMR (400 MHz, $CDCl_3$) δ 8.33 (d, *J* = 7.9 Hz, 1H), 7.37 (t, *J* = 7.8 Hz, 1H), 6.95 (d, *J* = 8.3 Hz, 1H), 6.89 (t, *J* = 7.5 Hz, 1H), 5.21 (brs, 2H), 3.22 (s, 3H), 3.17 (s, 3H).

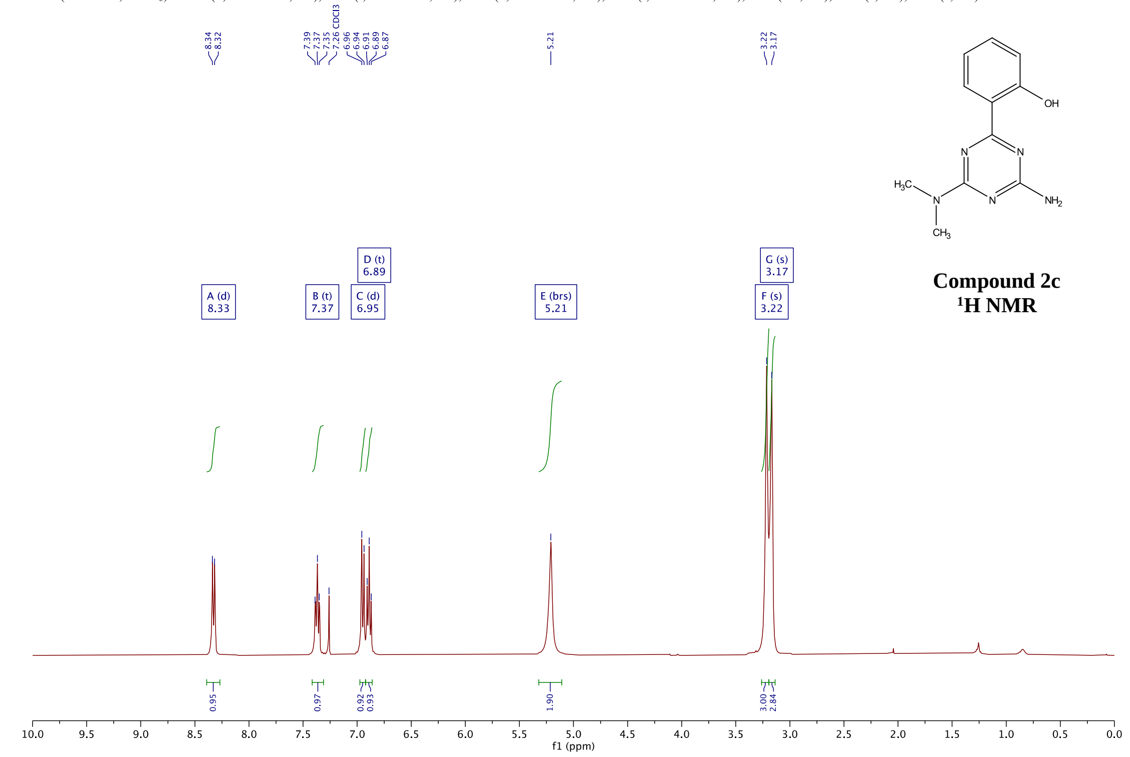

Figure 9. ^{1}H NMR of 2-(4-amino-6-(dimethylamino)-1,3,5-triazin-2-yl)phenol (**2c**)

^{13}C NMR (101 MHz, $CDCl_3$) δ 171.3, 165.8, 164.4, 162.2, 134.1, 129.7, 119.1, 118.4, 118.1, 36.9, 36.7.

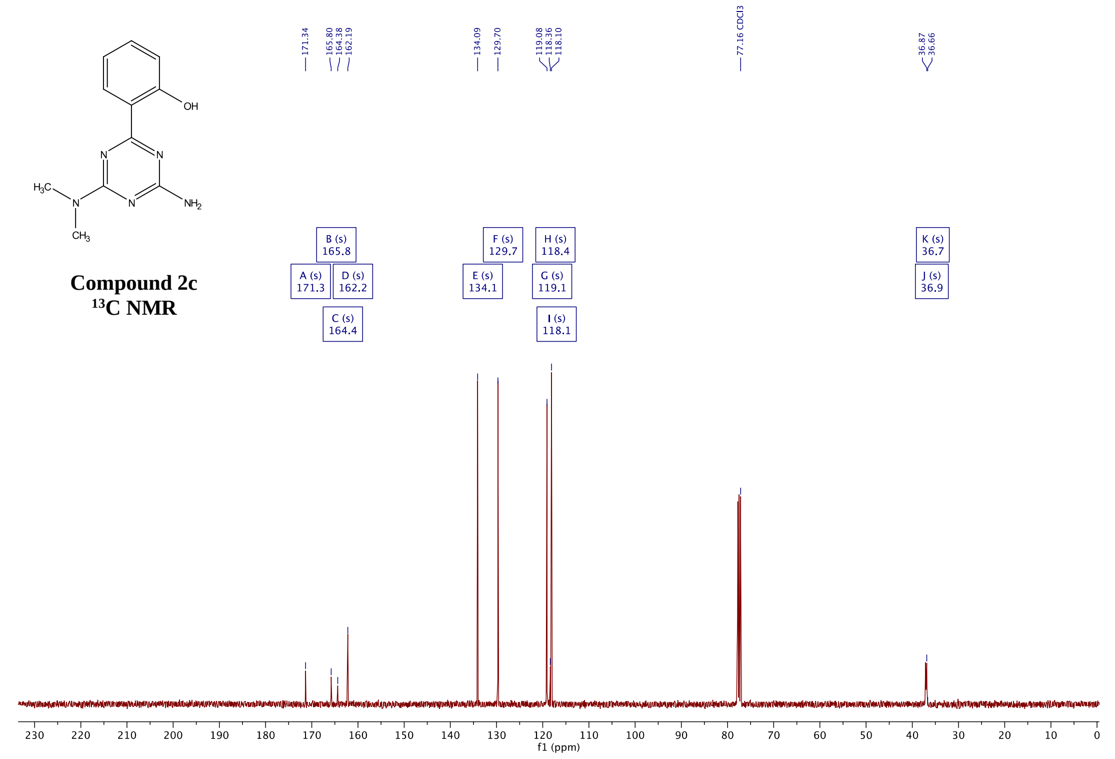

Figure 10. ^{13}C NMR of 2-(4-amino-6-(dimethylamino)-1,3,5-triazin-2-yl)phenol (**2c**)

Mass Spectrum List Report

Analysis Info

Analysis Name	D:\Data\Data Service\190819\A46_RC6_01_2982.d	Acquisition Date	8/19/2019 10:23:02 PM
Method	nv_pos_6min_profile_wguardcol_190624.m	Operator	CU.
Sample Name	A46	Instrument / Ser#	micrOTOF-Q II 10335
Comment			

Acquisition Parameter

Source Type	ESI	Ion Polarity	Positive	Set Nebulizer	3.0 Bar
Focus	Not active	Set Capillary	4000 V	Set Dry Heater	200 °C
Scan Begin	100 m/z	Set End Plate Offset	-500 V	Set Dry Gas	8.0 l/min
Scan End	1500 m/z	Set Collision Cell RF	250.0 Vpp	Set Divert Valve	Waste

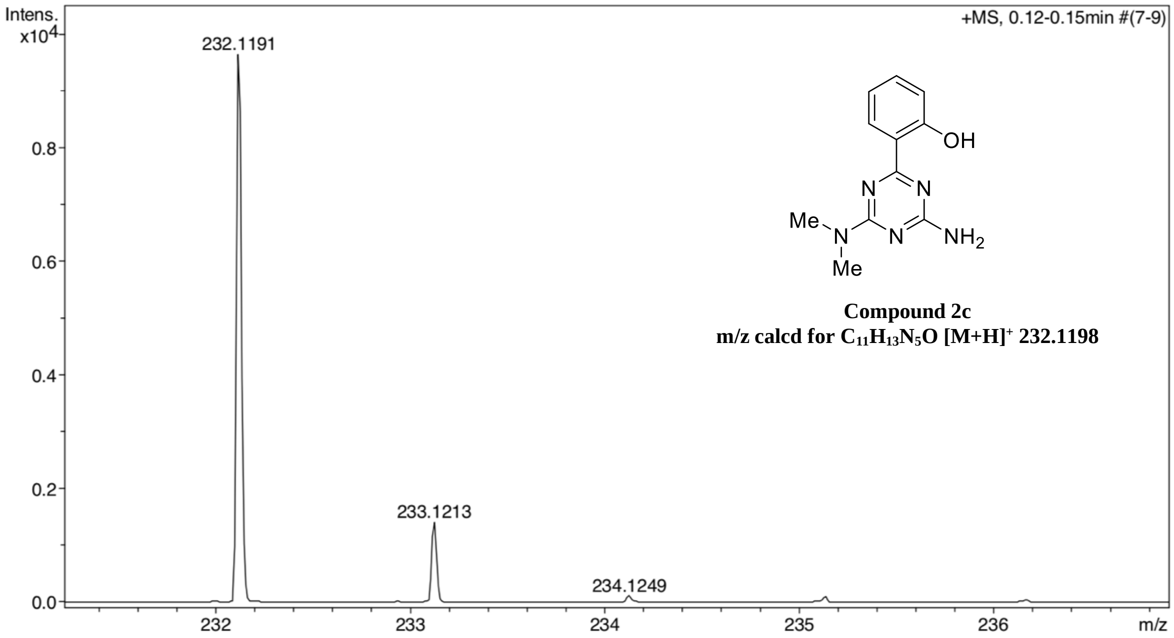

Figure 11. HRMS spectrum of 2-(4-amino-6-(dimethylamino)-1,3,5-triazin-2-yl)phenol (**2c**)

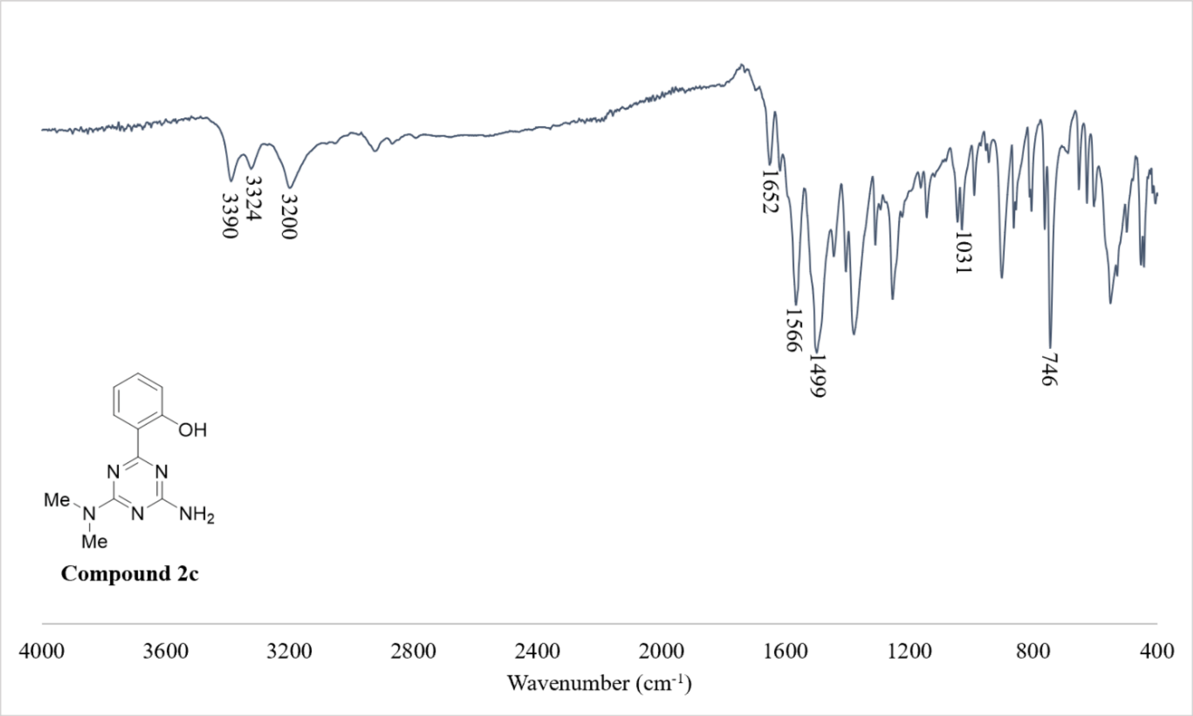

Figure 12. IR spectrum of 2-(4-amino-6-(dimethylamino)-1,3,5-triazin-2-yl)phenol (**2c**)

^{1}H NMR (400 MHz, $CDCl_3$) δ 7.95 (d, *J* = 15.9 Hz, 1H), 7.58 (d, *J* = 7.0 Hz, 2H), 7.43 – 7.30 (m, 3H), 6.83 (d, *J* = 15.9 Hz, 1H), 5.28 (brs, 2H), 3.22 (brs, 3H), 3.15 (brs, 3H).

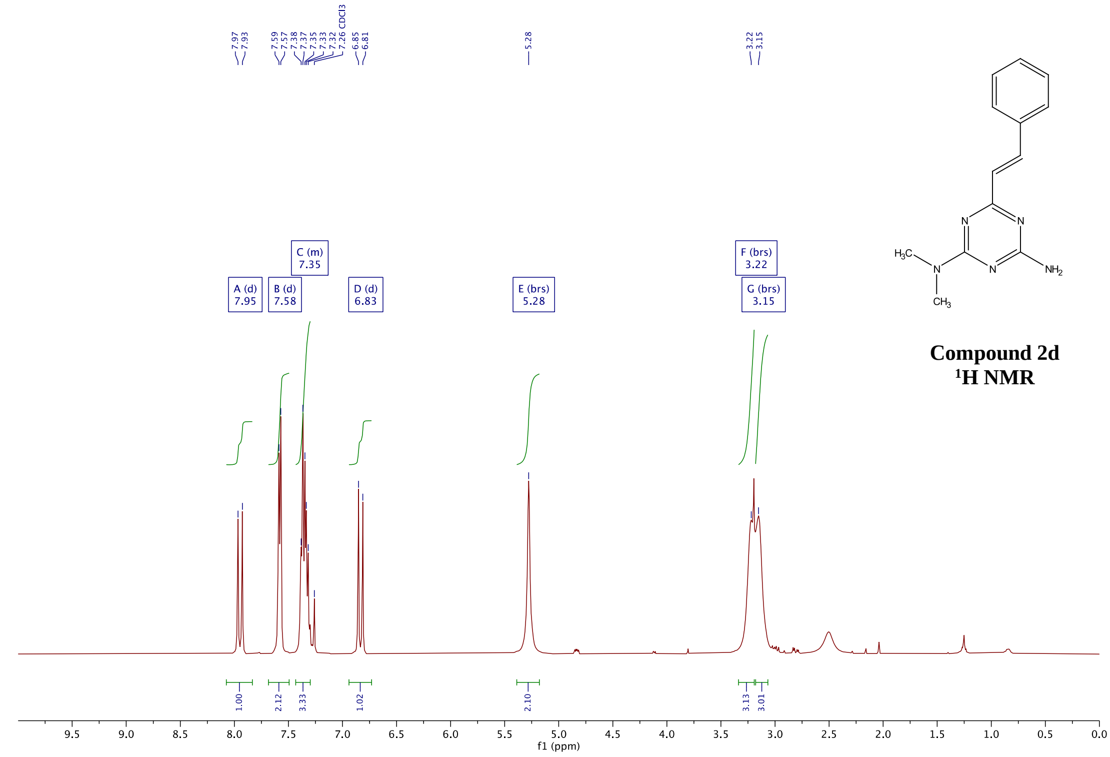

Figure 13. ^{1}H NMR of (*E*)-*N*^{2},*N*^{2}-dimethyl-6-styryl-1,3,5-triazine-2,4-diamine (**2d**)

^{13}C NMR (101 MHz, $CDCl_3$) δ 171.0, 167.2, 166.1, 139.7, 136.3, 129.6, 129.2, 128.3, 127.5, 36.7.

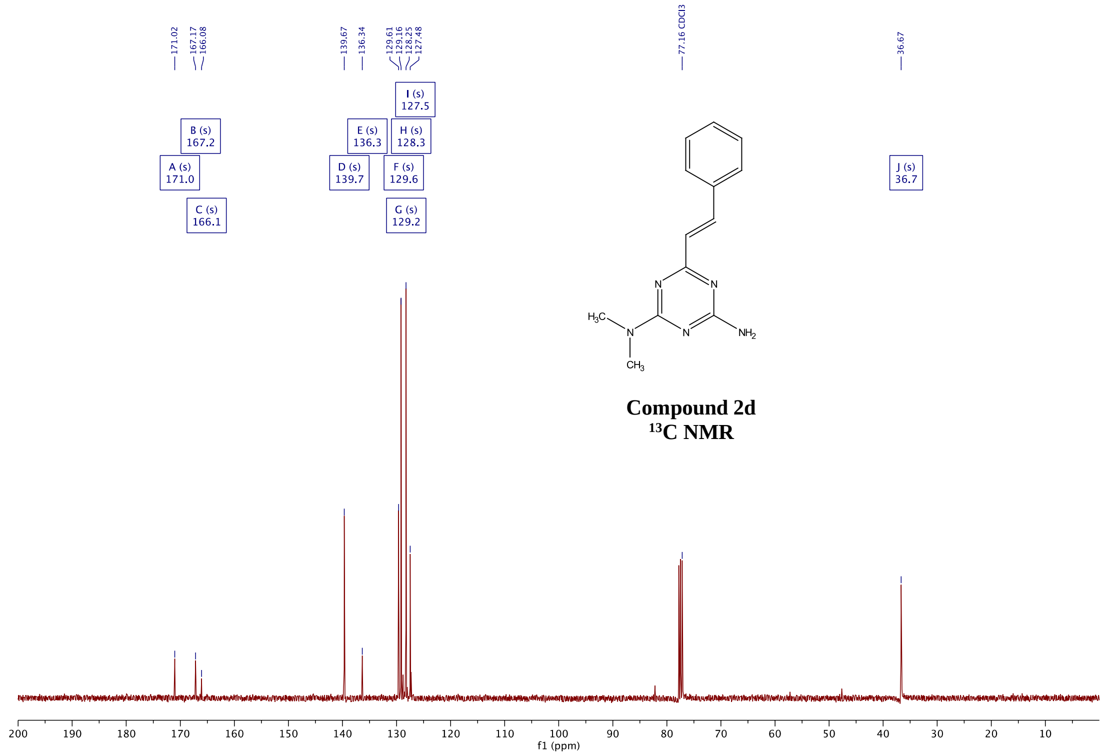

Figure 14. ^{13}C NMR of (*E*)-*N*^{2},*N*^{2}-dimethyl-6-styryl-1,3,5-triazine-2,4-diamine (**2d**)

^{1}H NMR (400 MHz, $CDCl_3$) δ 5.68 (brs, 2H), 3.95 (s, 3H), 3.23 (s, 3H), 3.13 (s, 3H).

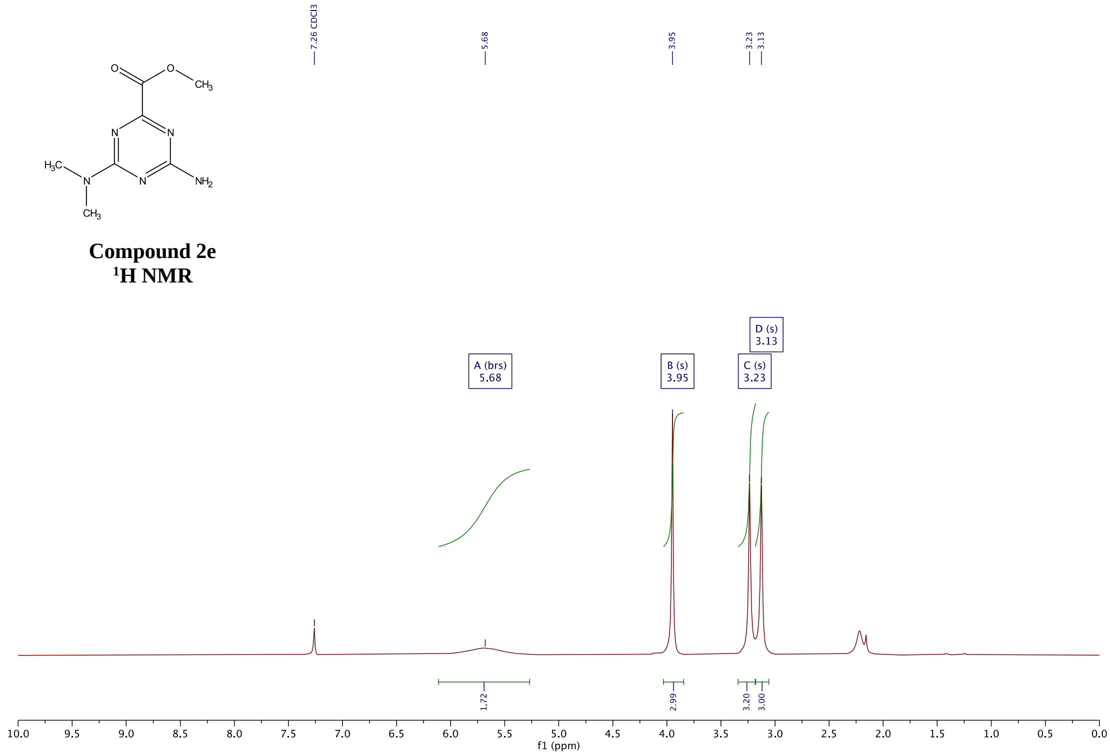

Figure 15. ^{1}H NMR of Methyl 4-amino-6-(dimethylamino)-1,3,5-triazine-2-carboxylate (**2e**)

^{13}C NMR (101 MHz, $CDCl_3$) δ 166.5, 165.4, 164.0, 163.1, 53.1, 36.4, 36.2.

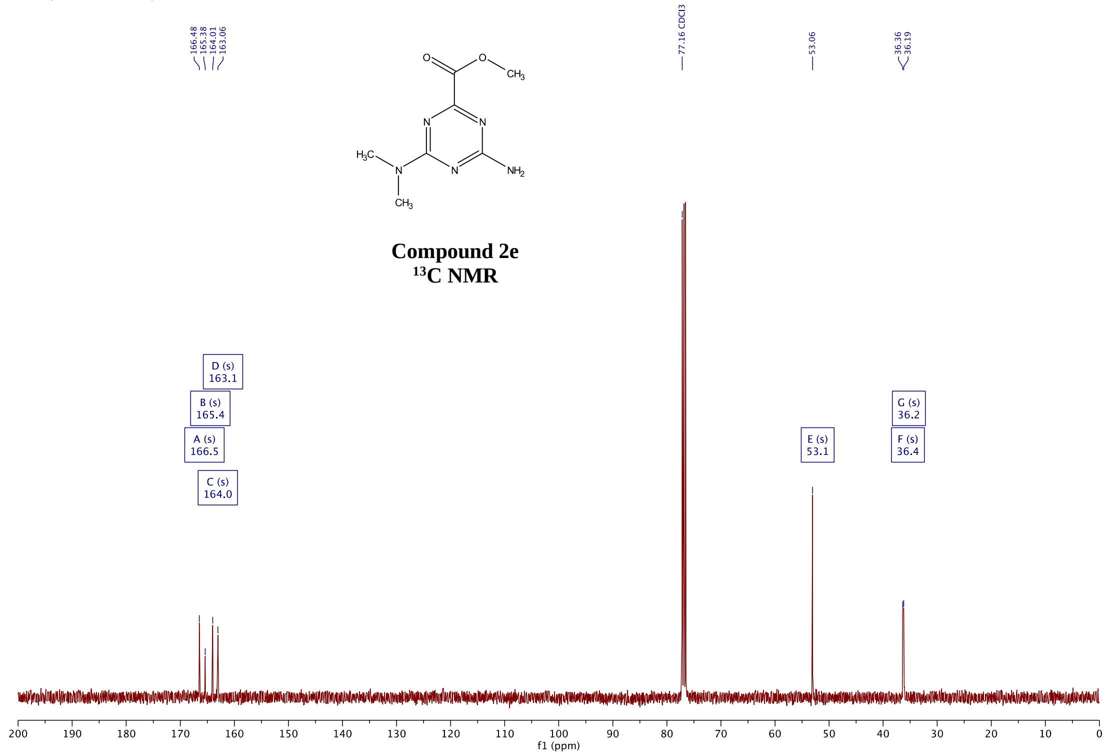

Figure 16. ^{13}C NMR of Methyl 4-amino-6-(dimethylamino)-1,3,5-triazine-2-carboxylate (**2e**)

Mass Spectrum List Report

Analysis Info

Analysis Name	D:\Data\Data Service\190819\A52_RC7_01_2983.d	Acquisition Date	8/19/2019 10:29:30 PM
Method	nv_pos_6min_profile_wguardcol_190624.m	Operator	CU.
Sample Name	A52	Instrument / Ser#	micrOTOF-Q II 10335
Comment			

Acquisition Parameter

Source Type	ESI	Ion Polarity	Positive	Set Nebulizer	3.0 Bar		
Focus	Not active	Set Capillary	4000 V	Set Dry Heater	200 ℃		
Scan Begin	100 m/z	Set End Plate Offset	-500 V	Set Dry Gas	8.0 l/min		
Scan End	1500 m/z	Set Collision Cell RF	250.0 Vpp	Set Divert Valve	Waste		

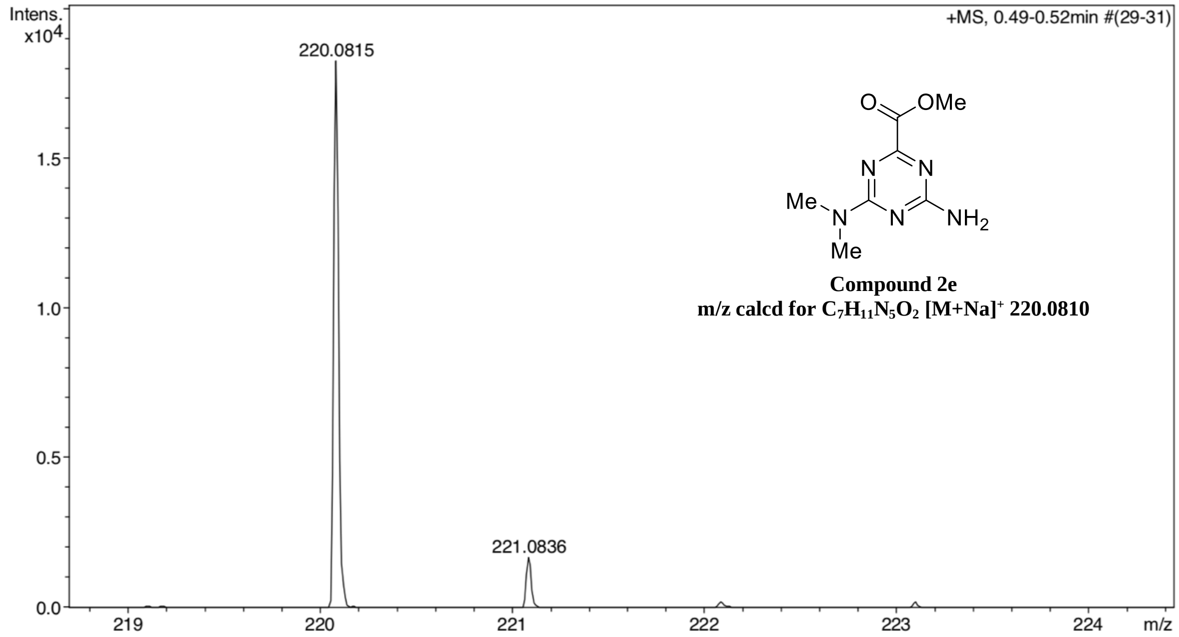

Figure 17. HRMS spectrum of Methyl 4-amino-6-(dimethylamino)-1,3,5-triazine-2-carboxylate (**2e**)

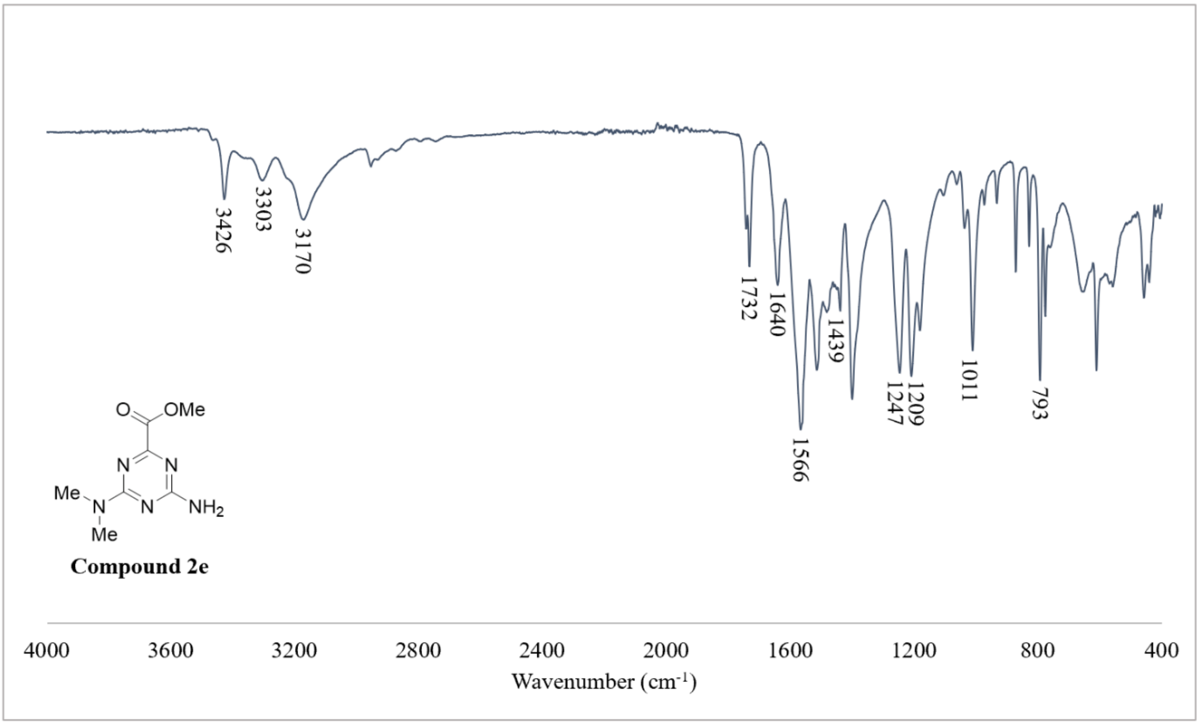

Figure 18. IR spectrum of Methyl 4-amino-6-(dimethylamino)-1,3,5-triazine-2-carboxylate (**2e**)

^{1}H NMR (400 MHz, DMSO) δ 11.08 (s, 1H), 9.01 (brs, 1H), 8.02 (brs, 1H), 3.11 (s, 3H), 3.02 (s, 3H).

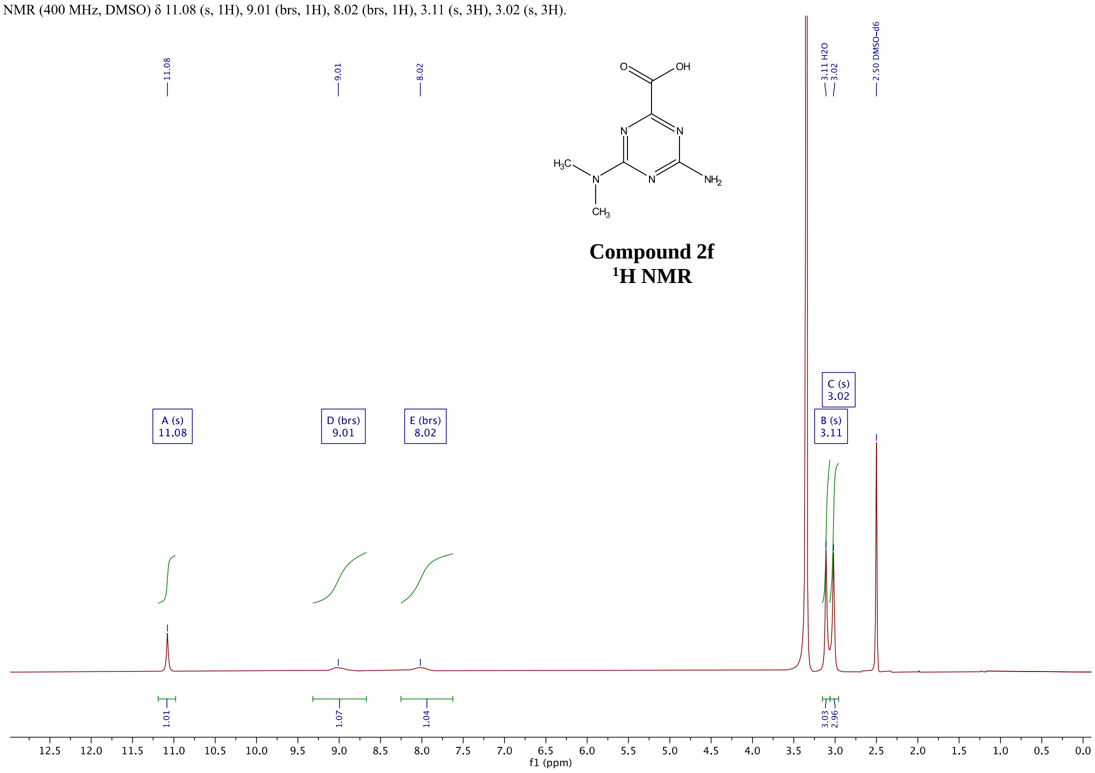

Figure 19. ^{1}H NMR of 4-amino-6-(dimethylamino)-1,3,5-triazine-2-carboxylic acid (**2f**)

^{13}C NMR (101 MHz, DMSO) δ 171.2, 169.9, 163.8, 160.4, 37.9, 37.2.

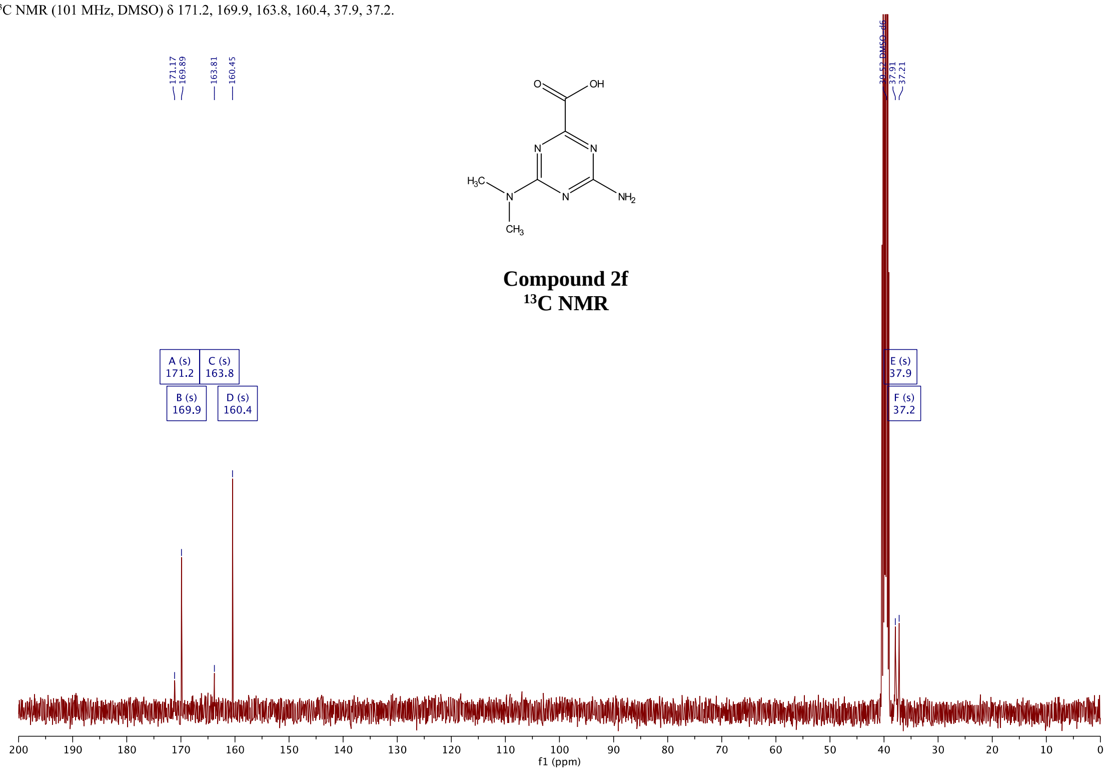

Figure 20. ^{13}C NMR of 4-amino-6-(dimethylamino)-1,3,5-triazine-2-carboxylic acid (**2f**)

Mass Spectrum List Report

Analysis Info

Analysis Name	D:\Data\Data Service\190819\A30_RA8_01_2948.d	Acquisition Date	8/19/2019 5:48:44 PM
Method	nv_pos_6min_profile_wguardcol_190624.m	Operator	CU.
Sample Name	A30	Instrument / Ser#	micrOTOF-Q II 10335
Comment			

Acquisition Parameter

Source Type	ESI	Ion Polarity	Positive	Set Nebulizer	3.0 Bar
Focus	Not active	Set Capillary	4000 V	Set Dry Heater	200 ℃
Scan Begin	100 m/z	Set End Plate Offset	-500 V	Set Dry Gas	8.0 l/min
Scan End	1500 m/z	Set Collision Cell RF	250.0 Vpp	Set Divert Valve	Waste

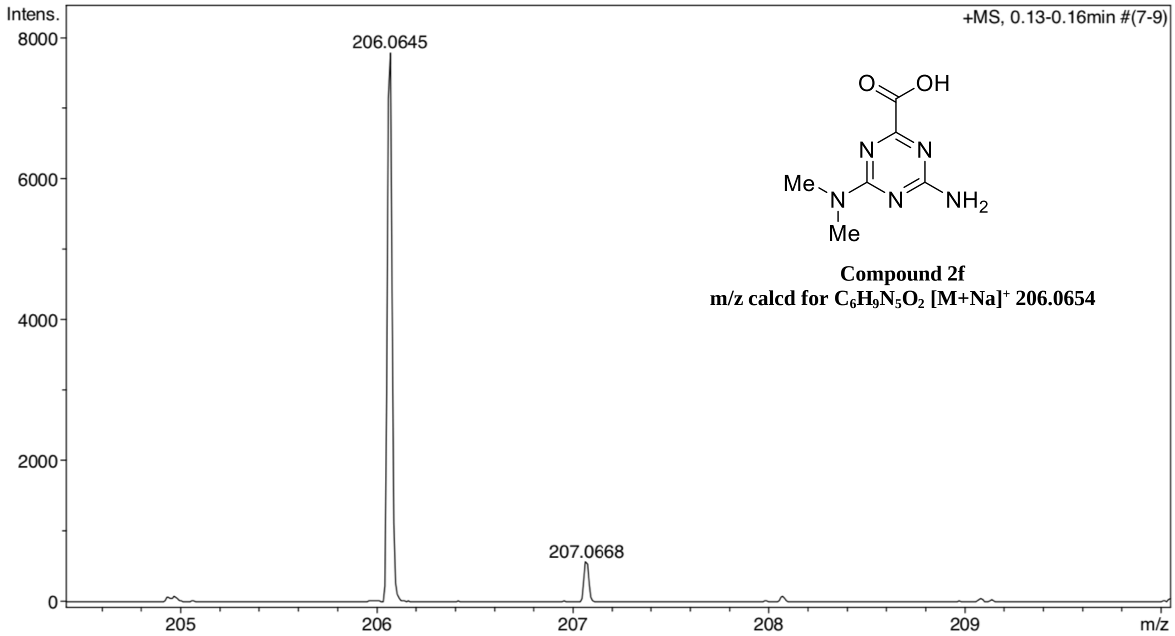

Figure 21. HRMS spectrum of 4-amino-6-(dimethylamino)-1,3,5-triazine-2-carboxylic acid (**2f**)

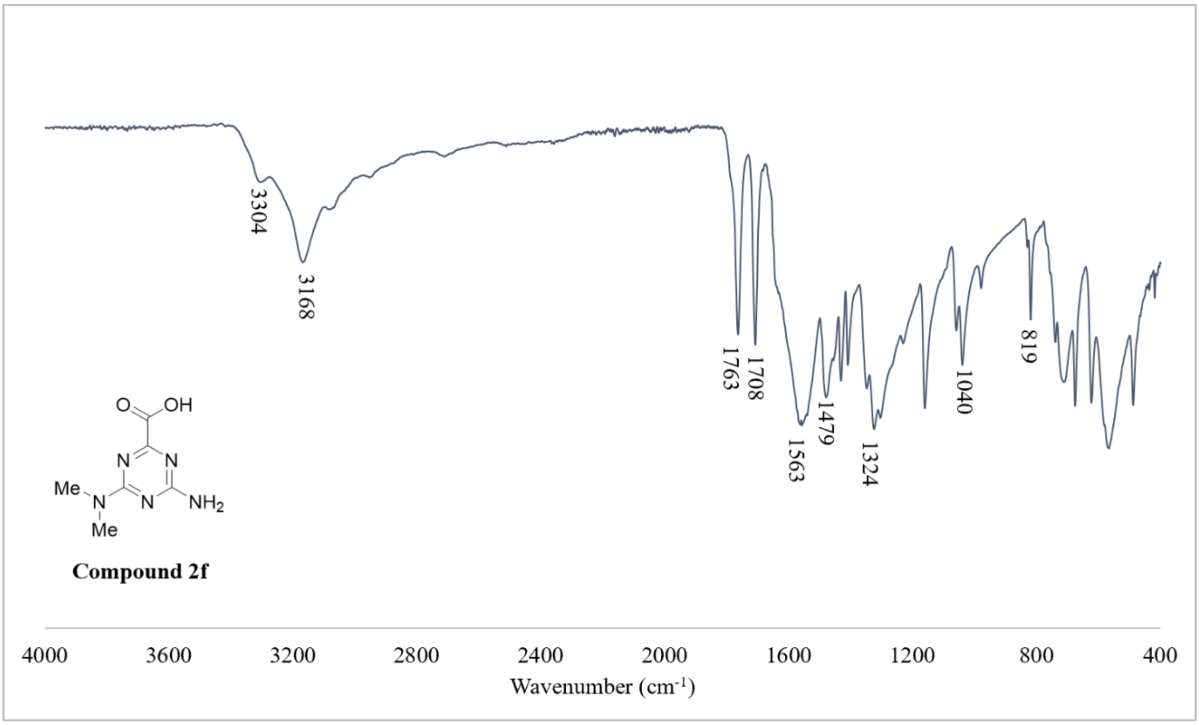

Figure 22. IR spectrum of 4-amino-6-(dimethylamino)-1,3,5-triazine-2-carboxylic acid (**2f**)

^{1}H NMR (400 MHz, DMSO) δ 9.38 (s, 1H), 7.77 (d, *J* = 7.9 Hz, 2H), 7.24 (t, *J* = 7.9 Hz, 2H), 6.94 (t, *J* = 7.2 Hz, 3H), 2.41 (t, *J* = 7.6 Hz, 2H), 1.73 – 1.60 (m, 2H), 1.29 – 1.20 (m, 23H), 0.84 (t, *J* = 6.7 Hz, 3H).

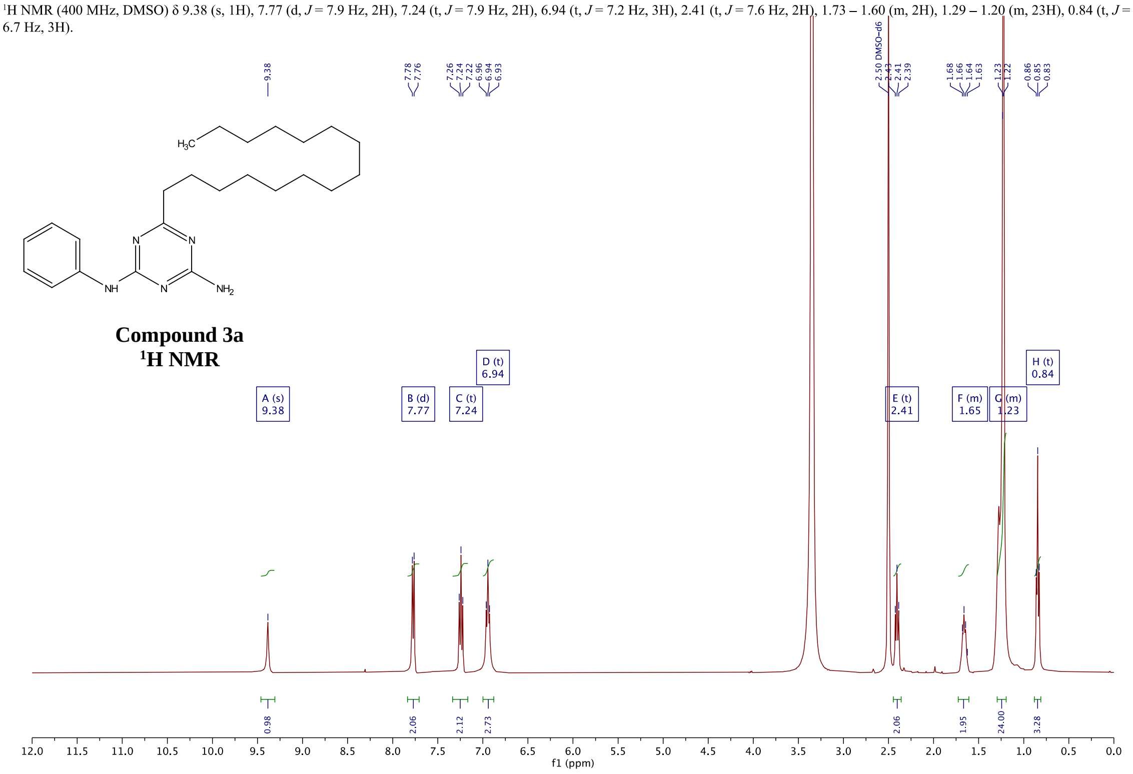

Figure 23. ^{1}H NMR of 6-pentadecyl-N^{2}-phenyl-1,3,5-triazine-2,4-diamine (**3a**)

^{13}C NMR (101 MHz, DMSO) δ 177.8, 166.6, 164.2, 140.0, 128.3, 121.7, 119.7, 37.9, 31.2, 29.0, 26.9, 22.0, 13.9.

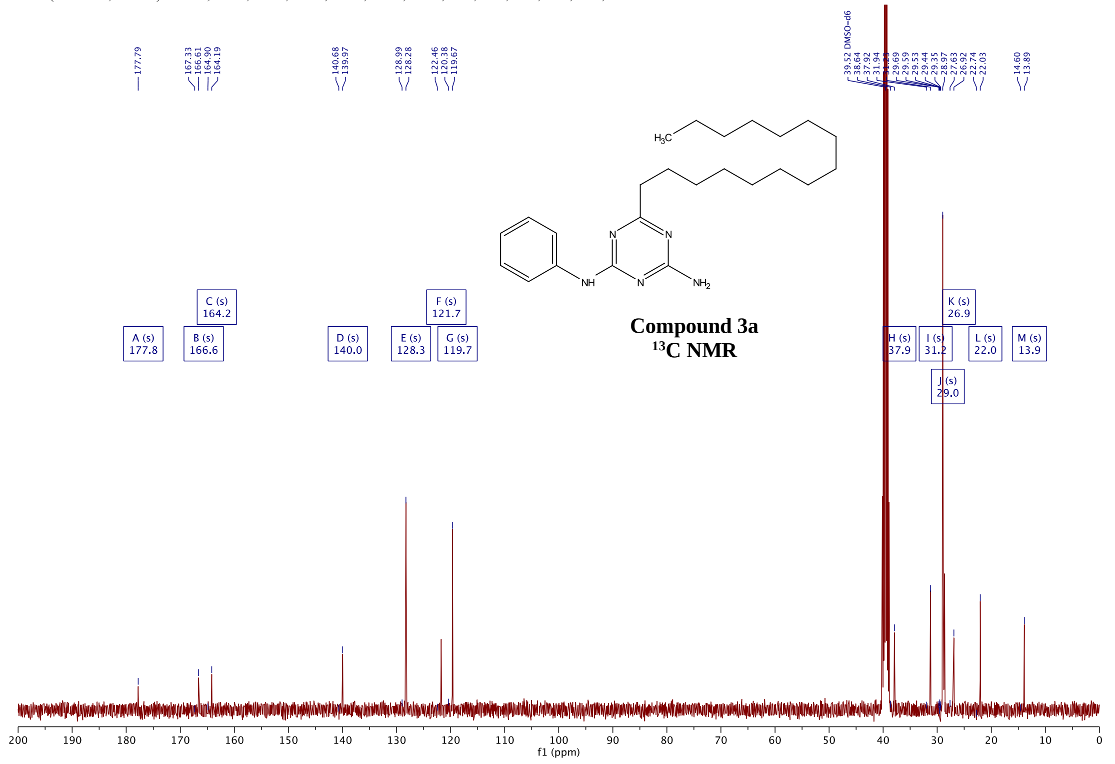

Figure 24. ^{13}C NMR of 6-pentadecyl-N^{2}-phenyl-1,3,5-triazine-2,4-diamine (**3a**)

Mass Spectrum List Report

Analysis Info

Analysis Name	D:\Data\Data Service\190819\A40_RC5_01_2981.d	Acquisition Date	8/19/2019 10:16:34 PM
Method	nv_pos_6min_profile_wguardcol_190624.m	Operator	CU.
Sample Name	A40	Instrument / Ser#	micrOTOF-Q II 10335
Comment			

Acquisition Parameter

Source Type	ESI	Ion Polarity	Positive	Set Nebulizer	3.0 Bar
Focus	Not active	Set Capillary	4000 V	Set Dry Heater	200 °C
Scan Begin	100 m/z	Set End Plate Offset	-500 V	Set Dry Gas	8.0 l/min
Scan End	1500 m/z	Set Collision Cell RF	250.0 Vpp	Set Divert Valve	Waste

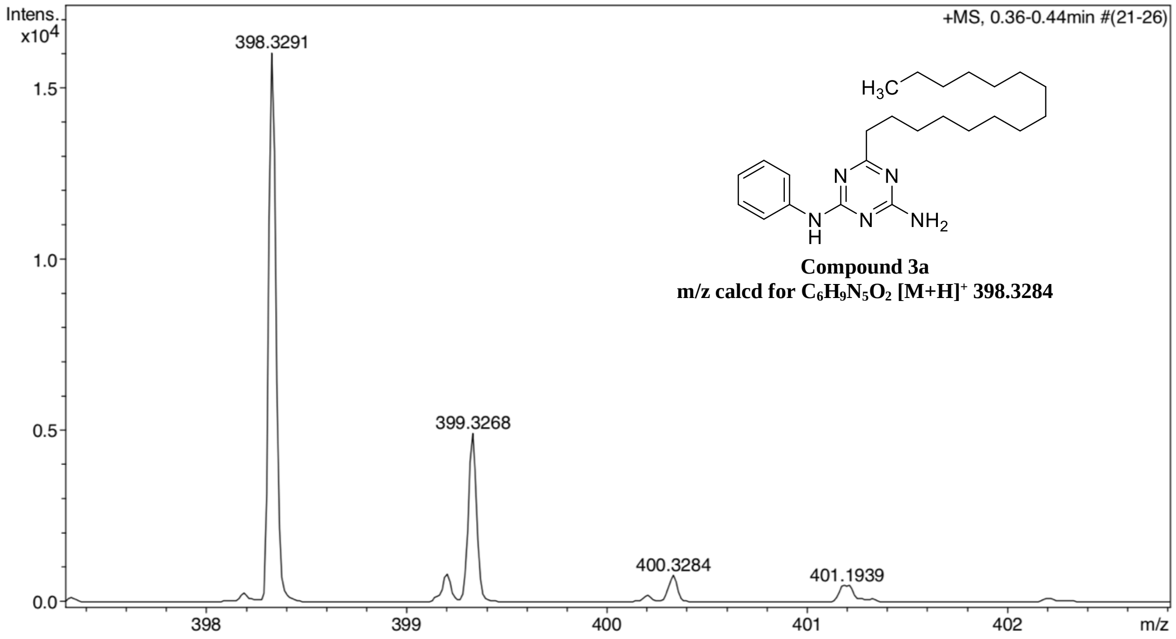

Figure 25. HRMS spectrum of 6-pentadecyl-*N*[2]-phenyl-1,3,5-triazine-2,4-diamine (**3a**)

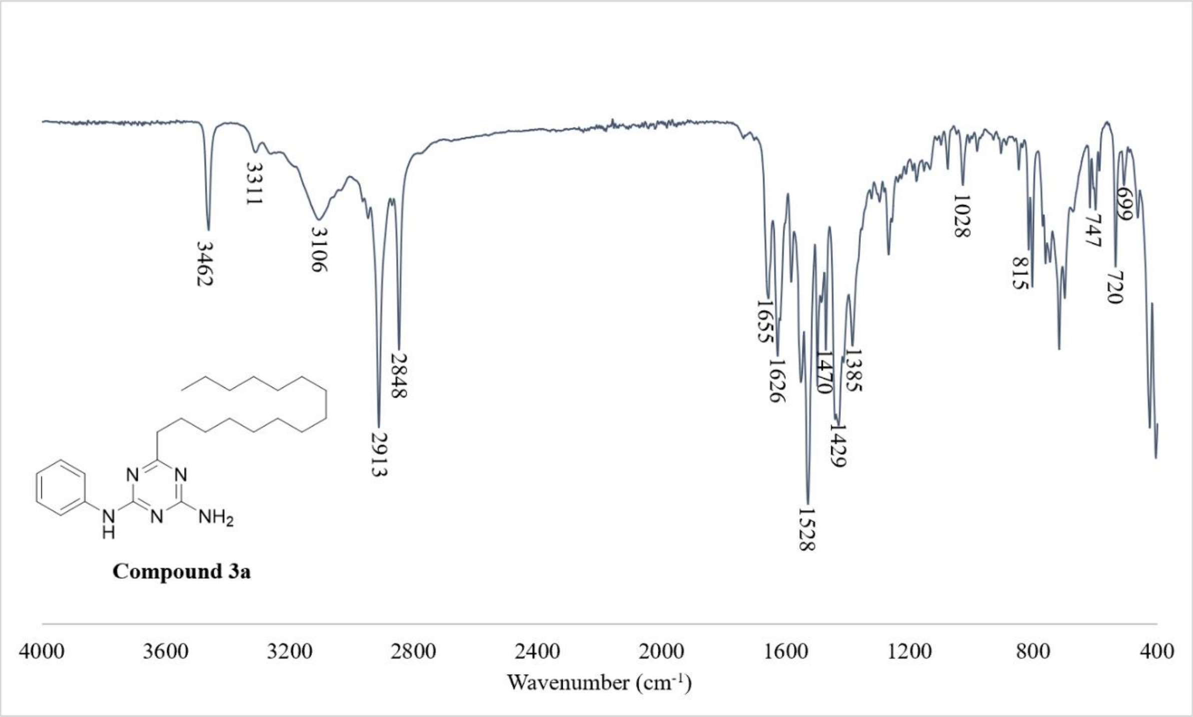

Figure 26. IR spectrum of 6-pentadecyl-*N*[2]-phenyl-1,3,5-triazine-2,4-diamine (**3a**)

^{1}H NMR (400 MHz, DMSO) δ 9.52 (s, 1H), 8.31 (d, J = 6.8 Hz, 2H), 7.84 (d, J = 8.0 Hz, 2H), 7.58 – 7.44 (m, 3H), 7.31 (t, J = 7.8 Hz, 2H), 7.12 (brs, 2H), 6.99 (t, J = 7.3 Hz, 1H).

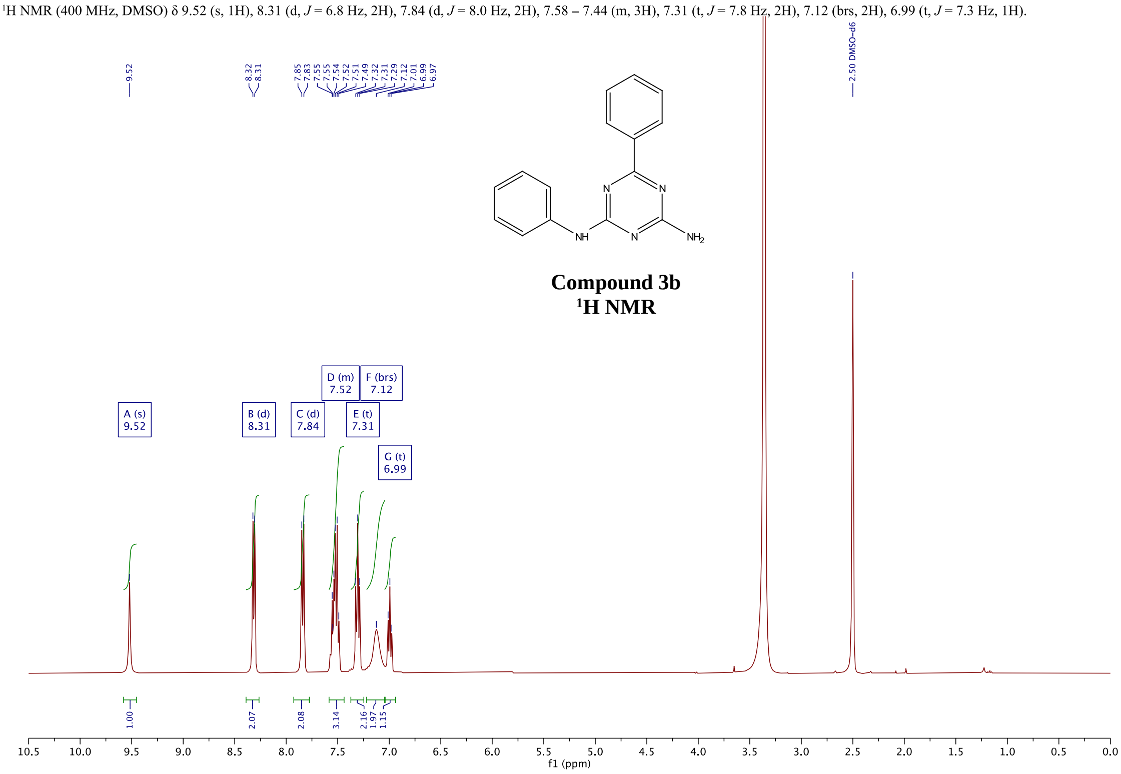

Figure 27. ^{1}H NMR of N^{2},6-diphenyl-1,3,5-triazine-2,4-diamine (**3b**)

^{13}C NMR (101 MHz, DMSO) δ 170.7, 167.6, 165.1, 140.3, 137.2, 131.8, 128.9, 128.7, 128.2, 122.5, 120.4.

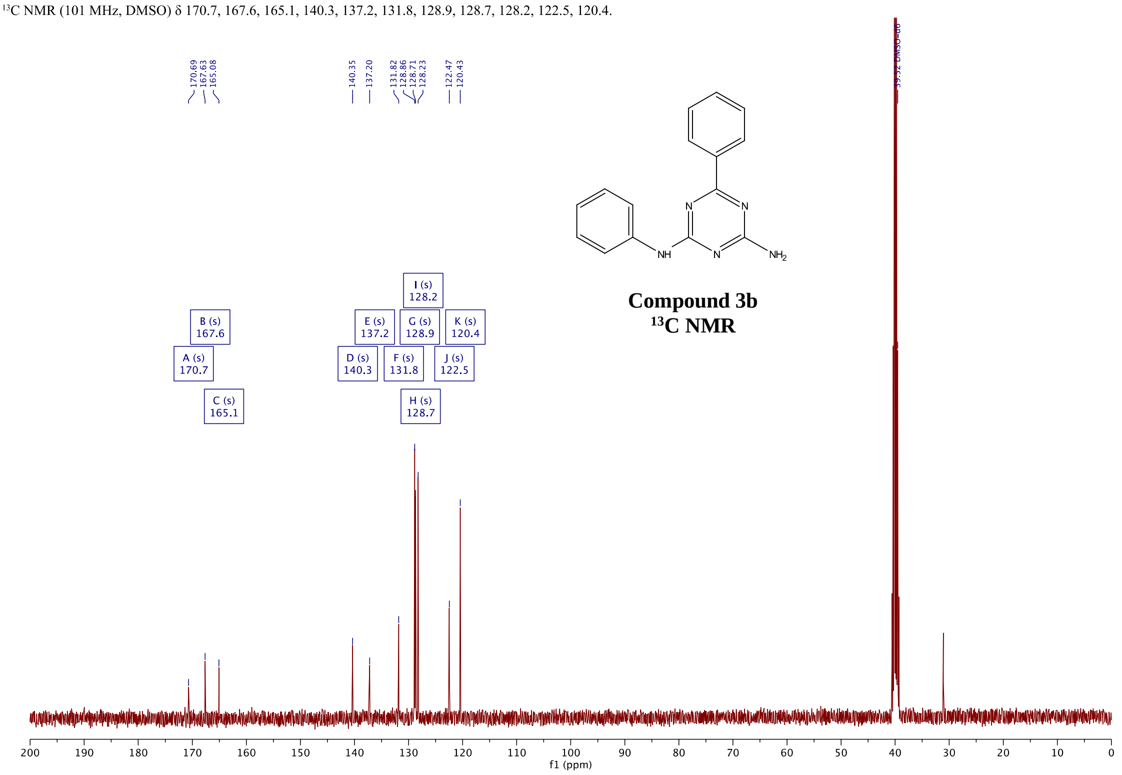

Figure 28. ^{13}C NMR of N^{2},6-diphenyl-1,3,5-triazine-2,4-diamine (**3b**)

^{1}H NMR (400 MHz, DMSO) δ 13.49 (s, 1H), 9.77 (brs, 1H), 8.26 (d, *J* = 7.3 Hz, 1H), 7.76 (brs, 2H), 7.55 (brs, 1H), 7.44 – 7.35 (m, 2H), 7.33 (t, *J* = 7.2 Hz, 2H), 7.04 (t, *J* = 7.5 Hz, 1H), 6.90 (t, *J* = 8.0 Hz, 2H).

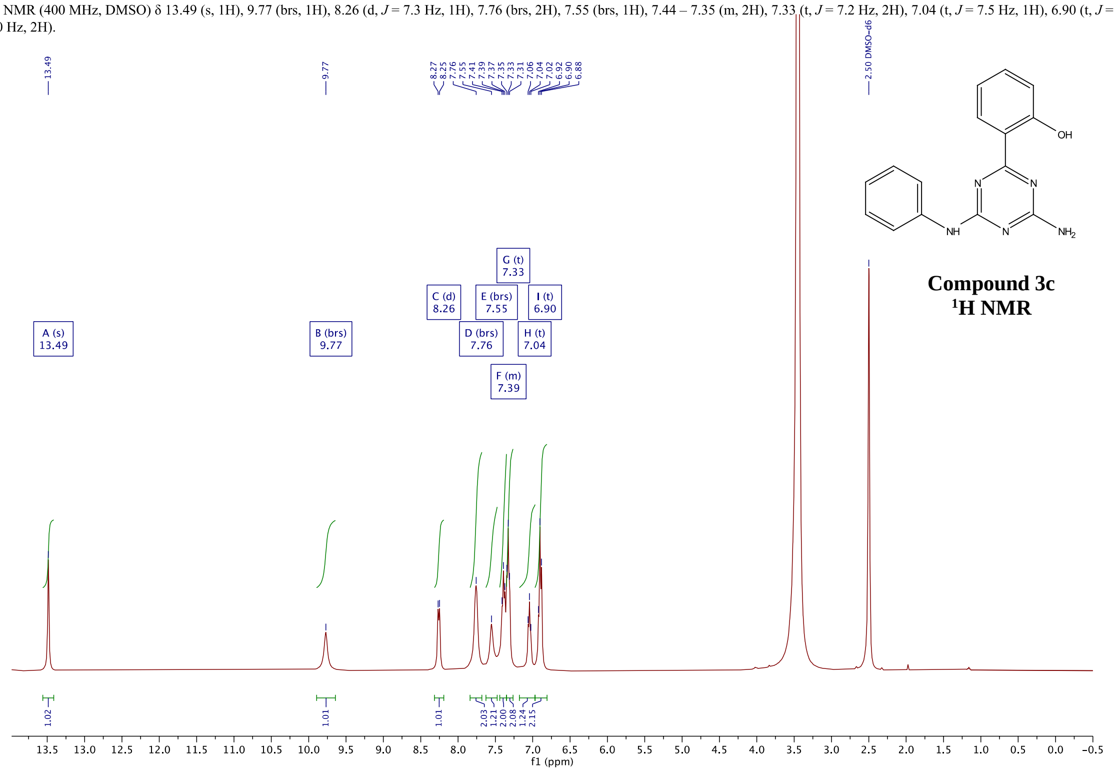

Figure 29. ^{1}H NMR of 2-(4-amino-6-(phenylamino)-1,3,5-triazin-2-yl)phenol (**3c**)

^{13}C NMR (101 MHz, DMSO) δ 169.9, 164.7, 160.8, 138.8, 133.2, 128.3, 128.1, 122.3, 120.2, 117.9, 117.2, 117.1.

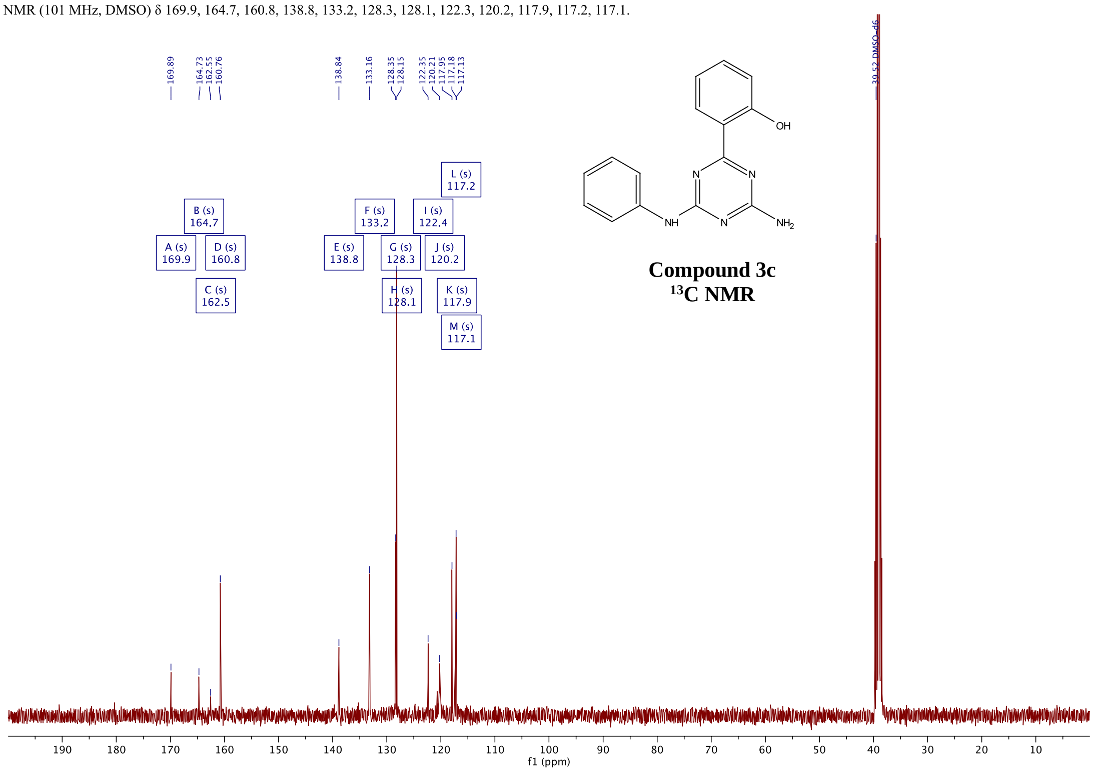

Figure 30. ^{13}C NMR of 2-(4-amino-6-(phenylamino)-1,3,5-triazin-2-yl)phenol (**3c**)

Mass Spectrum List Report

Analysis Info

Analysis Name	D:\Data\Data Service\190819\A38_RC4_01_2980.d	Acquisition Date	8/19/2019 10:10:06 PM
Method	nv_pos_6min_profile_wguardcol_190624.m	Operator	CU.
Sample Name	A38	Instrument / Ser#	micrOTOF-Q II 10335
Comment			

Acquisition Parameter

Source Type	ESI	Ion Polarity	Positive	Set Nebulizer	3.0 Bar
Focus	Not active	Set Capillary	4000 V	Set Dry Heater	200 °C
Scan Begin	100 m/z	Set End Plate Offset	-500 V	Set Dry Gas	8.0 l/min
Scan End	1500 m/z	Set Collision Cell RF	250.0 Vpp	Set Divert Valve	Waste

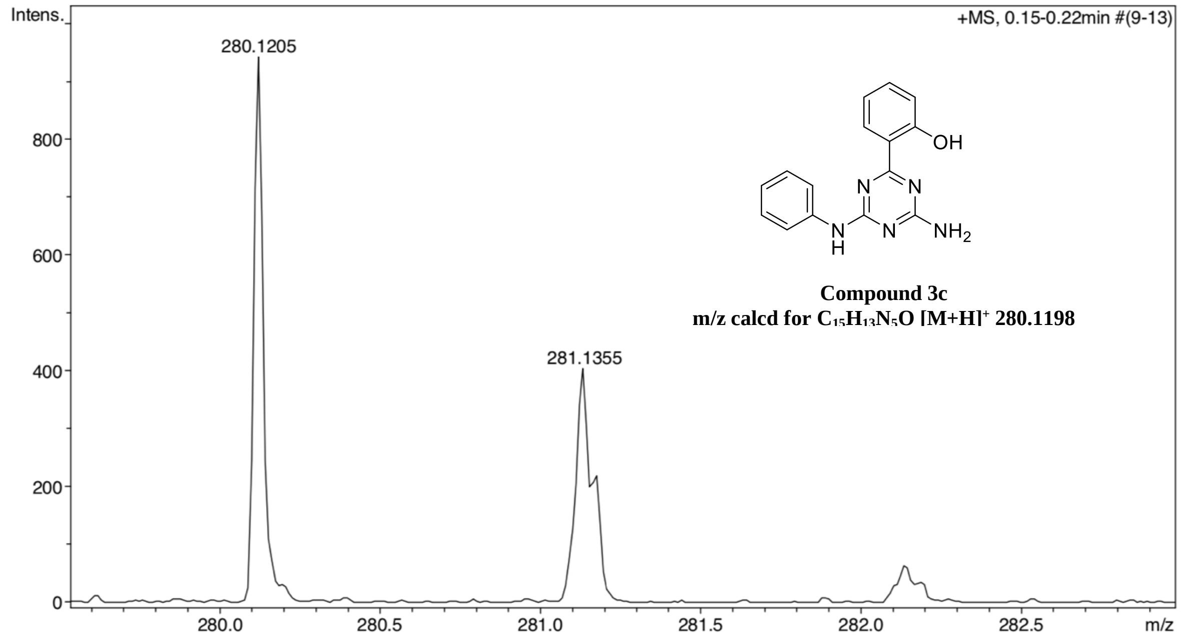

Figure 31. HRMS spectrum of 2-(4-amino-6-(phenylamino)-1,3,5-triazin-2-yl)phenol (**3c**)

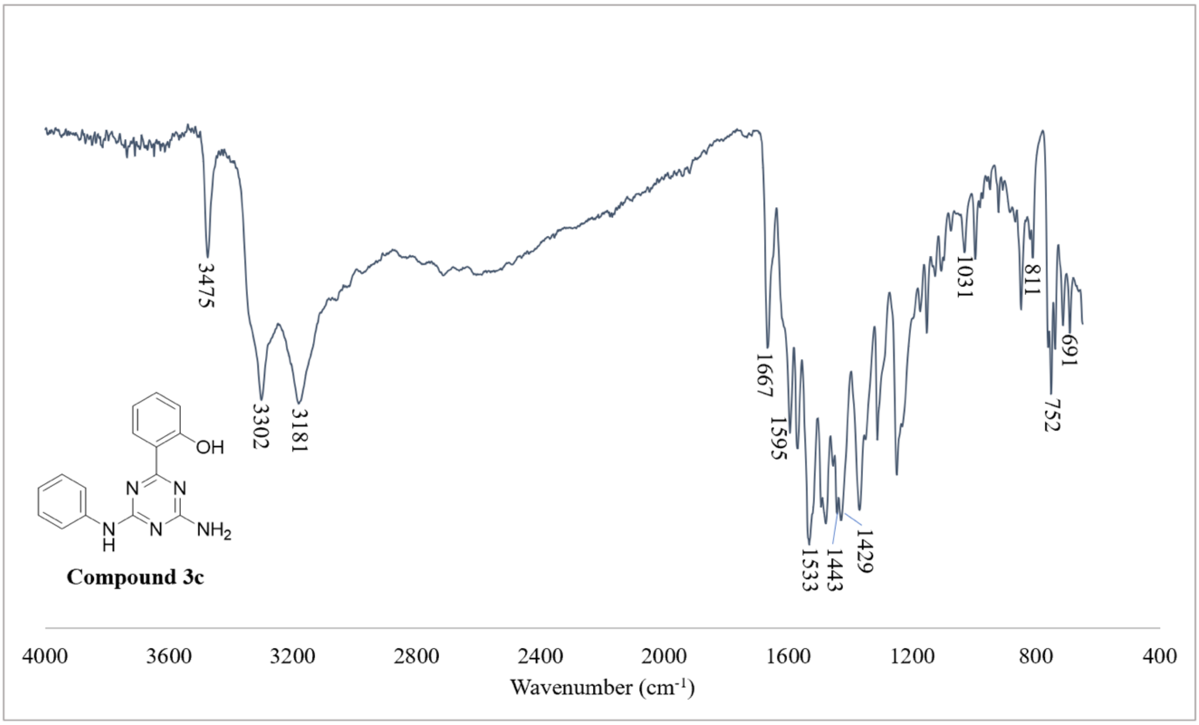

Figure 32. IR spectrum of 2-(4-amino-6-(phenylamino)-1,3,5-triazin-2-yl)phenol (**3c**)

^{1}H NMR (500 MHz,) δ 9.47 (s, 1H), 7.87 (d, J = 15.9 Hz, 1H), 7.81 (dd, J = 8.7, 1.2 Hz, 2H), 7.66 (d, J = 7.5 Hz, 2H), 7.46 – 7.36 (m, 3H), 7.28 (t, J = 7.6 Hz, 2H), 7.03 (brs, 2H), 6.97 (tt, J = 7.3, 1.2 Hz, 1H), 6.82 (d, J = 16.0 Hz, 1H).

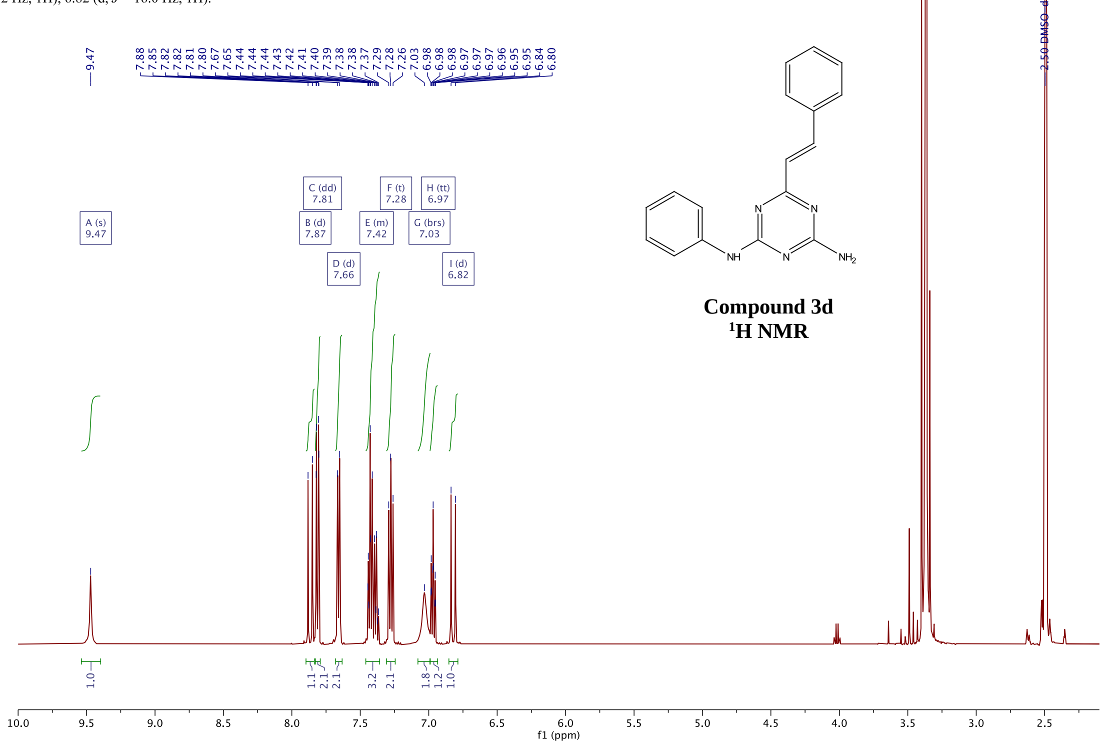

Figure 33. ^{1}H NMR of (E)-N^{2}-phenyl-6-styryl-1,3,5-triazine-2,4-diamine (**3d**)

^{13}C NMR (101 MHz, $CDCl_3$) δ 171.0, 166.6, 164.3, 141.2, 138.3, 135.5, 129.9, 129.1, 129.0, 128.2, 125.5, 124.0, 121.0.

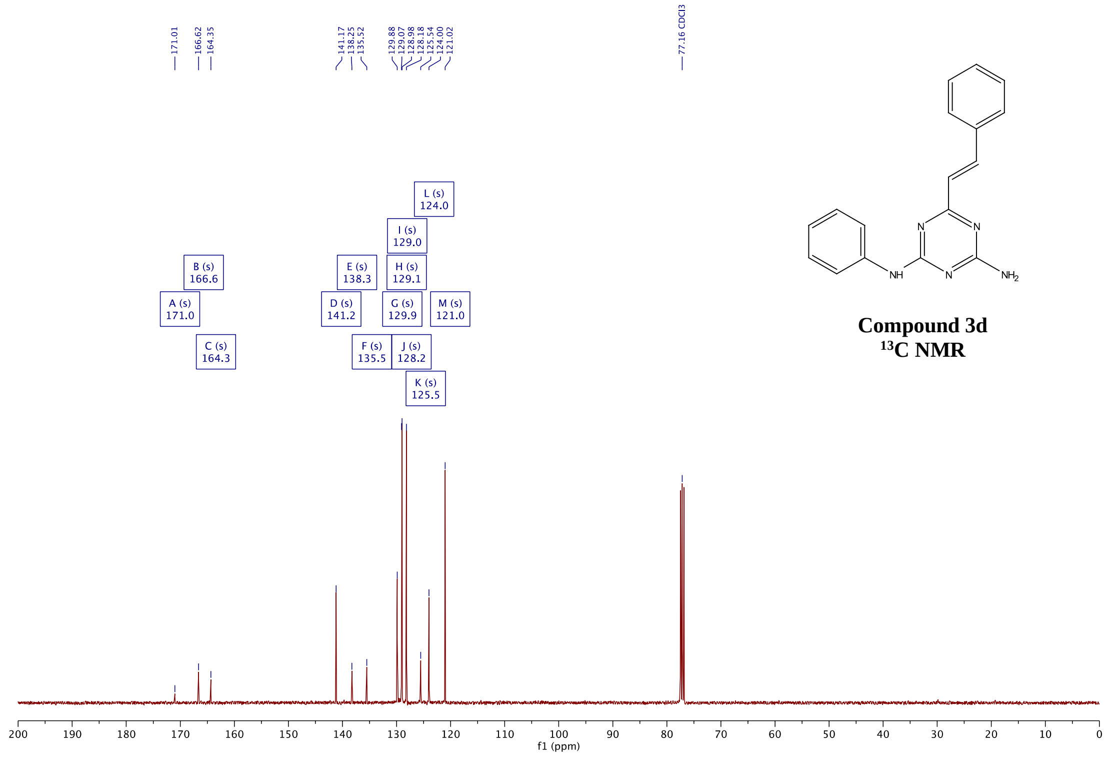

Figure 34. ^{13}C NMR of (E)-N^{2}-phenyl-6-styryl-1,3,5-triazine-2,4-diamine (**3d**)

Mass Spectrum List Report

Analysis Info

Analysis Name	D:\Data\Data Service\190819\A37_RC3_01_2979.d	Acquisition Date	8/19/2019 10:03:39 PM
Method	nv_pos_6min_profile_wguardcol_190624.m	Operator	CU.
Sample Name	A37	Instrument / Ser#	micrOTOF-Q II 10335
Comment			

Acquisition Parameter

Source Type	ESI	Ion Polarity	Positive	Set Nebulizer	3.0 Bar
Focus	Not active	Set Capillary	4000 V	Set Dry Heater	200 ℃
Scan Begin	100 m/z	Set End Plate Offset	-500 V	Set Dry Gas	8.0 l/min
Scan End	1500 m/z	Set Collision Cell RF	250.0 Vpp	Set Divert Valve	Waste

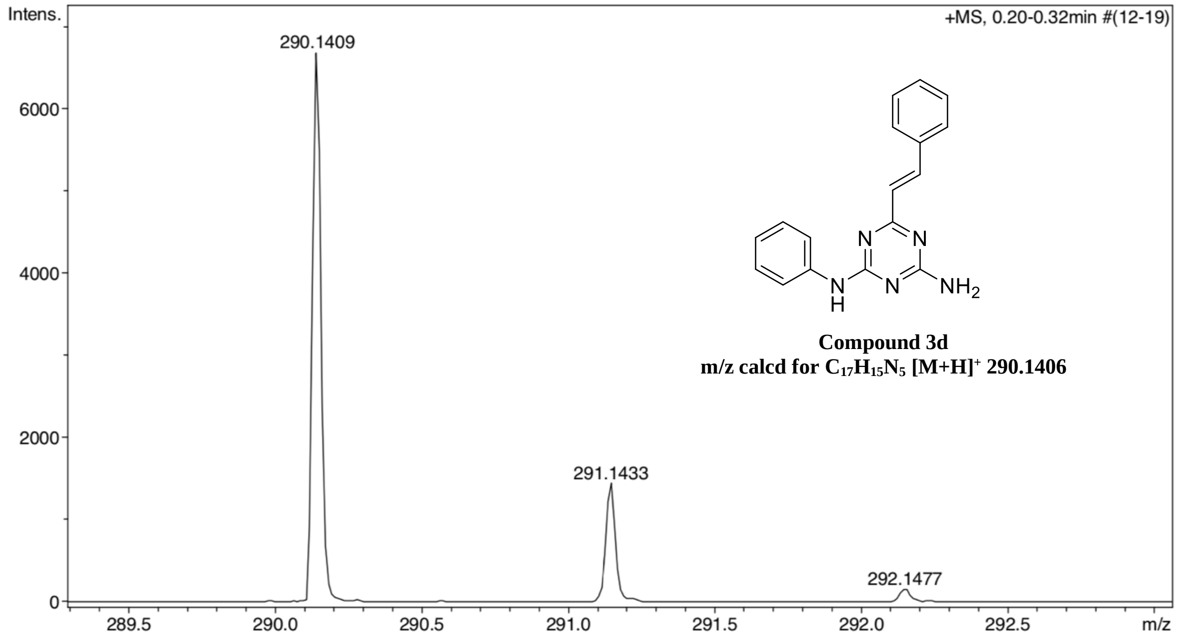

Figure 35. HRMS spectrum of (*E*)-*N*^{2}-phenyl-6-styryl-1,3,5-triazine-2,4-diamine (**3d**)

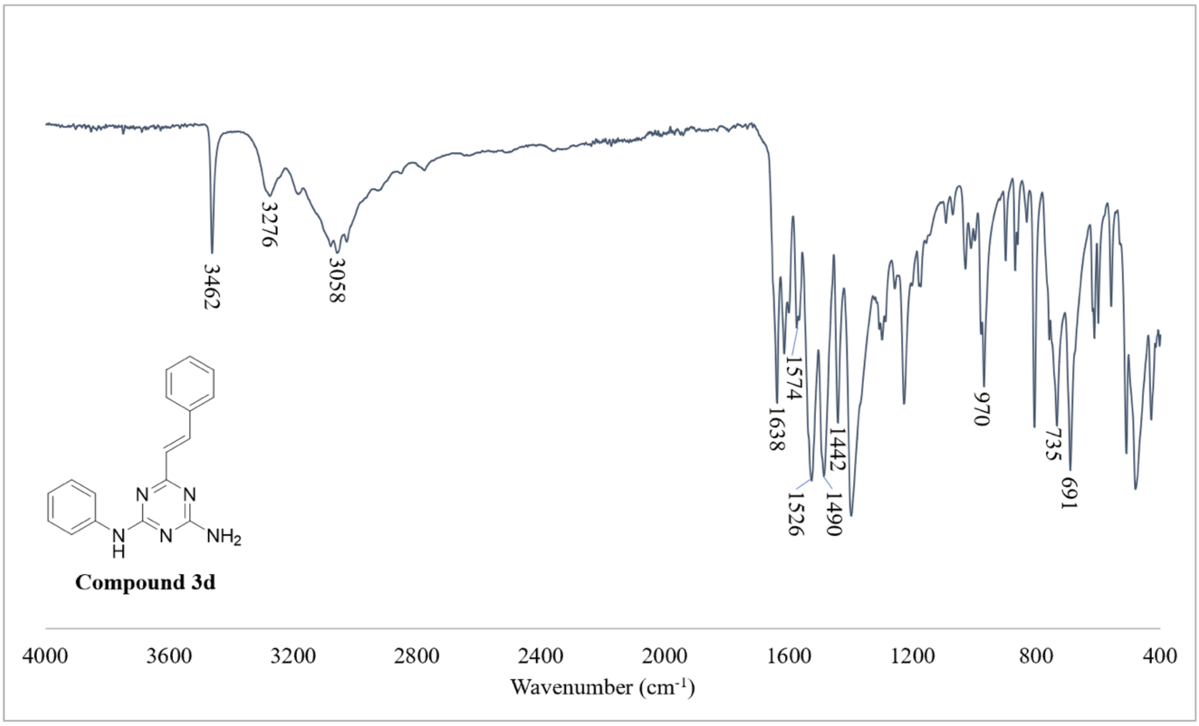

Figure 36. IR spectrum of (*E*)-*N*[2]-phenyl-6-styryl-1,3,5-triazine-2,4-diamine (**3d**)

¹H NMR (400 MHz, DMSO) δ 9.93 (brs, 1H), 7.78 (d, *J* = 7.9 Hz, 2H), 7.56 (brs, 1H), 7.43 (brs, 1H), 7.28 (t, *J* = 6.8 Hz, 2H), 7.01 (t, *J* = 7.9 Hz, 1H), 3.83 (s, 3H).

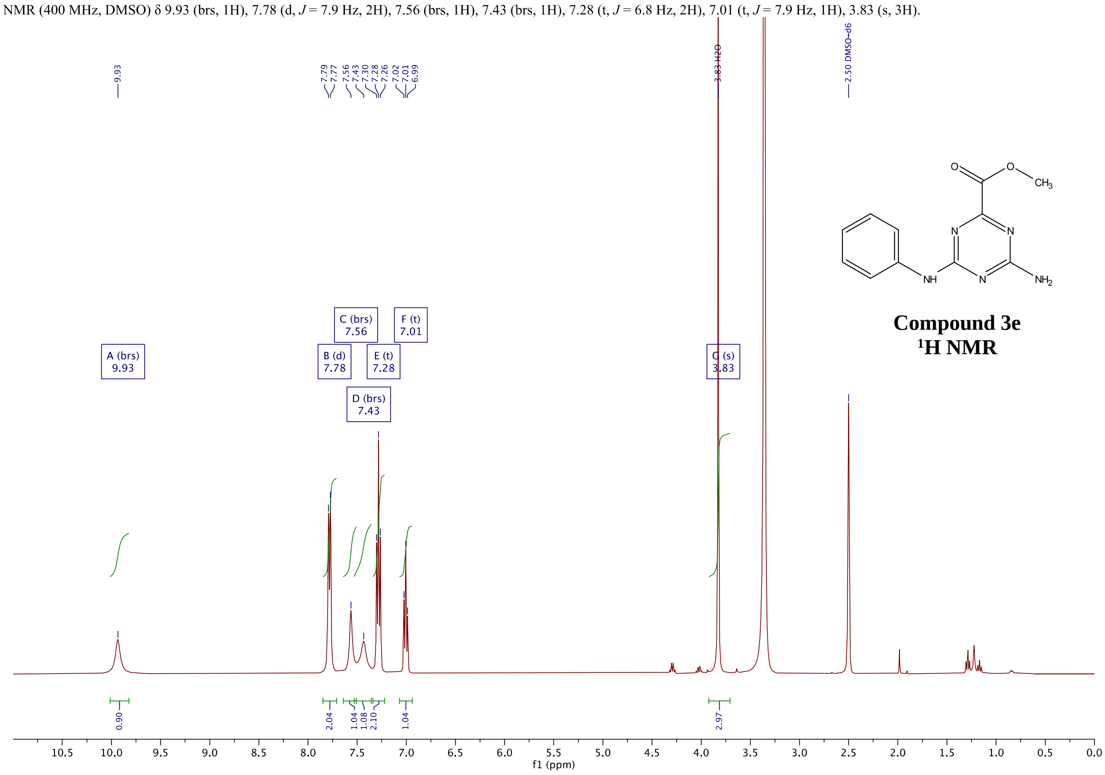

Figure 37. ¹H NMR of Methyl 4-amino-6-(phenylamino)-1,3,5-triazine-2-carboxylate (**3e**)

¹³C NMR (101 MHz, DMSO) δ 167.3, 164.5, 164.2, 164.1, 139.5, 128.7, 122.7, 120.3, 52.6.

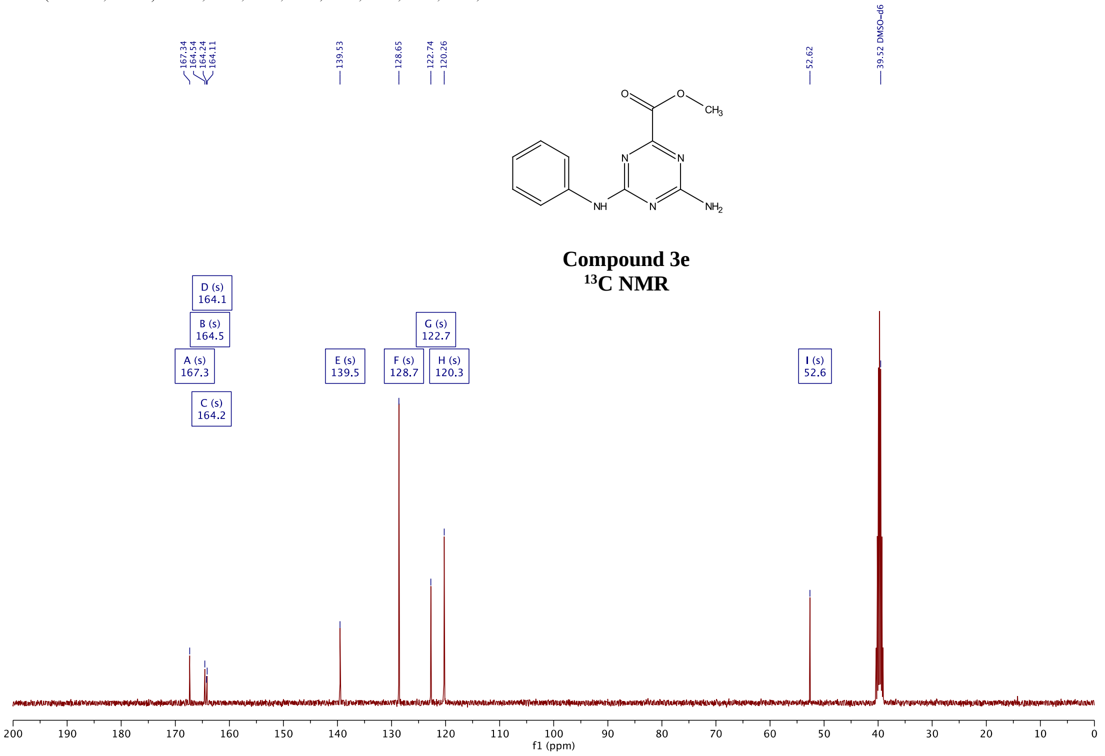

Figure 38. ¹³C NMR of Methyl 4-amino-6-(phenylamino)-1,3,5-triazine-2-carboxylate (**3e**)

^{1}H NMR (400 MHz, DMSO) δ 9.89 (brs, 1H), 7.78 (d, *J* = 8.1 Hz, 2H), 7.47 (brs, 2H), 7.28 (t, *J* = 7.3 Hz, 2H), 7.00 (t, *J* = 7.5, 6.7 Hz, 1H).

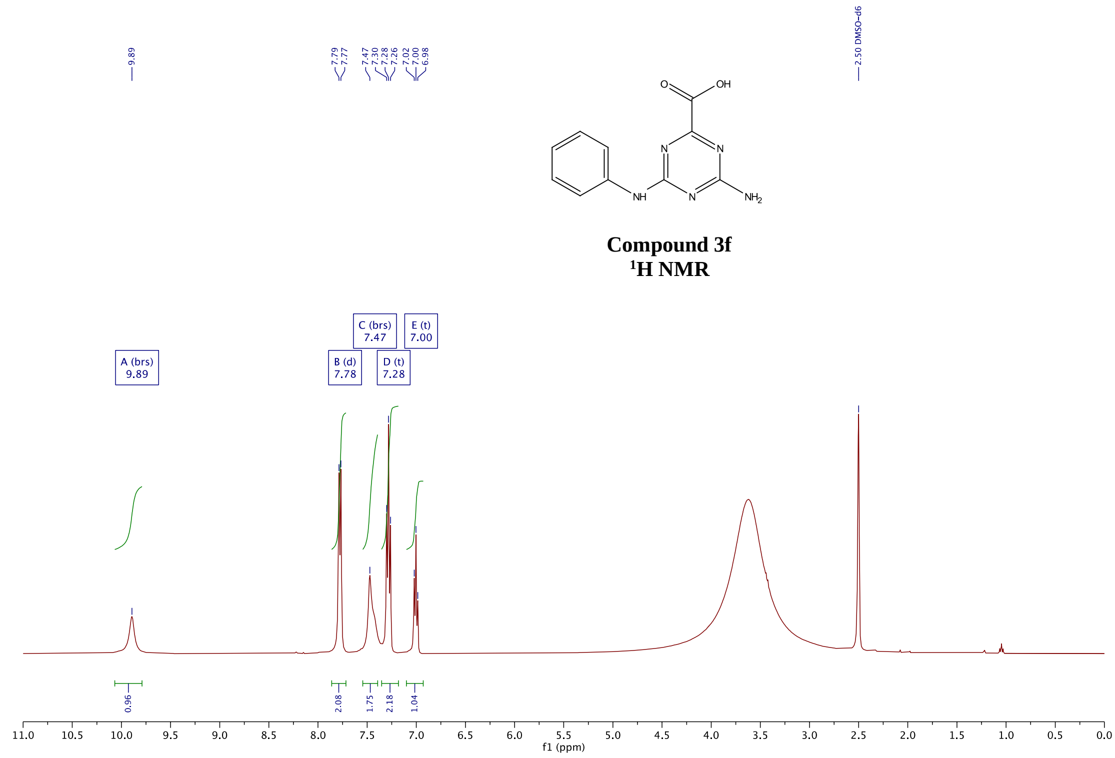

Figure 39. ^{1}H NMR of 4-amino-6-(phenylamino)-1,3,5-triazine-2-carboxylic acid (**3f**)

^{13}C NMR (101 MHz, DMSO) δ 166.9, 165.4, 165.0, 164.3, 139.4, 128.5, 122.6, 120.2.

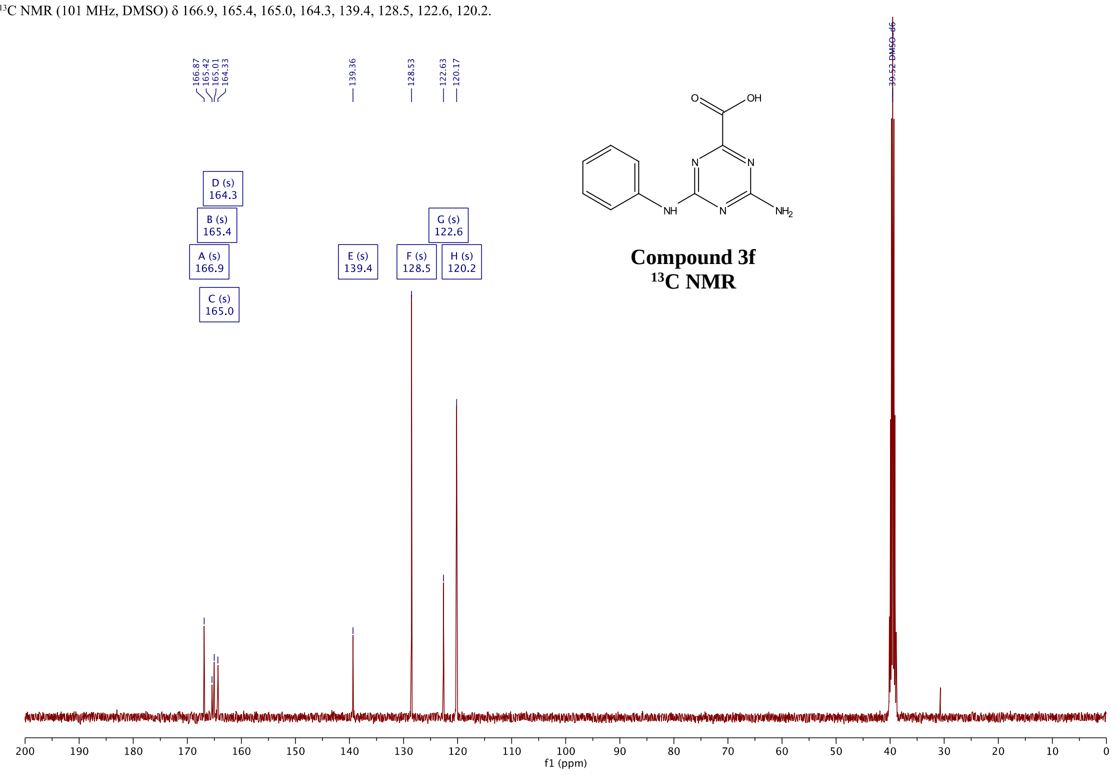

Figure 40. ^{13}C NMR of 4-amino-6-(phenylamino)-1,3,5-triazine-2-carboxylic acid (**3f**)

Mass Spectrum List Report

Analysis Info

Analysis Name	D:\Data\Data Service\190827\A53_RB1_01_3017.d	Acquisition Date	8/27/2019 9:33:05 PM
Method	nv_pos_6min_profile_wguardcol_190624.m	Operator	CU.
Sample Name	A53	Instrument / Ser#	micrOTOF-Q II 10335
Comment			

Acquisition Parameter

Source Type	ESI	Ion Polarity	Positive	Set Nebulizer	3.0 Bar
Focus	Not active	Set Capillary	4000 V	Set Dry Heater	200 °C
Scan Begin	100 m/z	Set End Plate Offset	-500 V	Set Dry Gas	8.0 l/min
Scan End	1500 m/z	Set Collision Cell RF	250.0 Vpp	Set Divert Valve	Waste

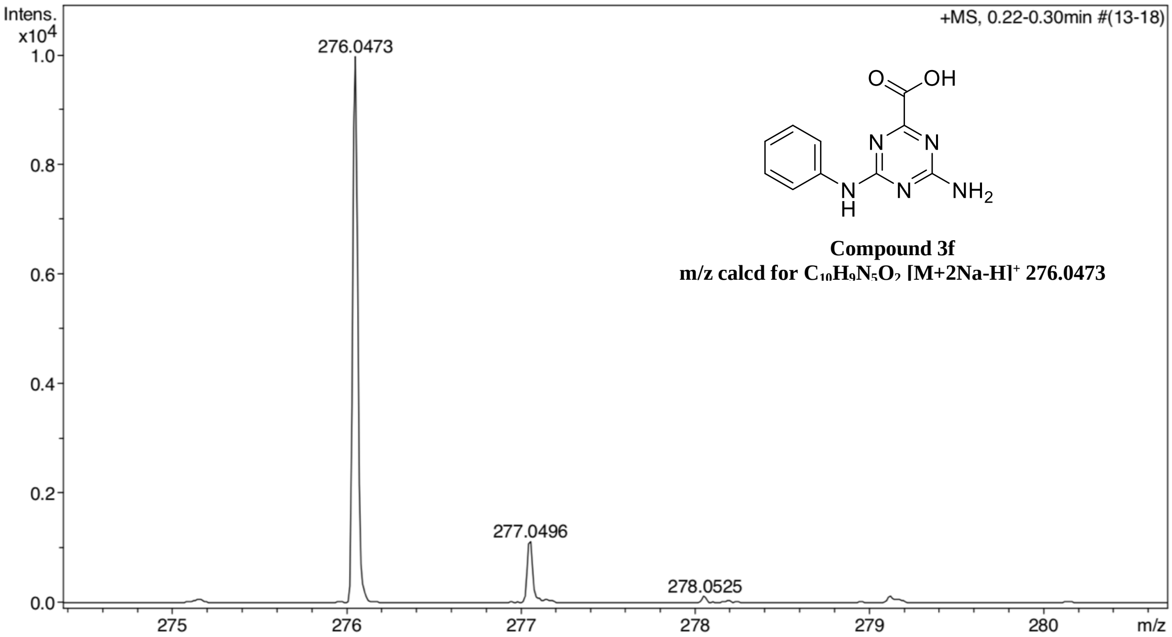

Figure 41. HRMS spectrum of 4-amino-6-(phenylamino)-1,3,5-triazine-2-carboxylic acid (**3f**)

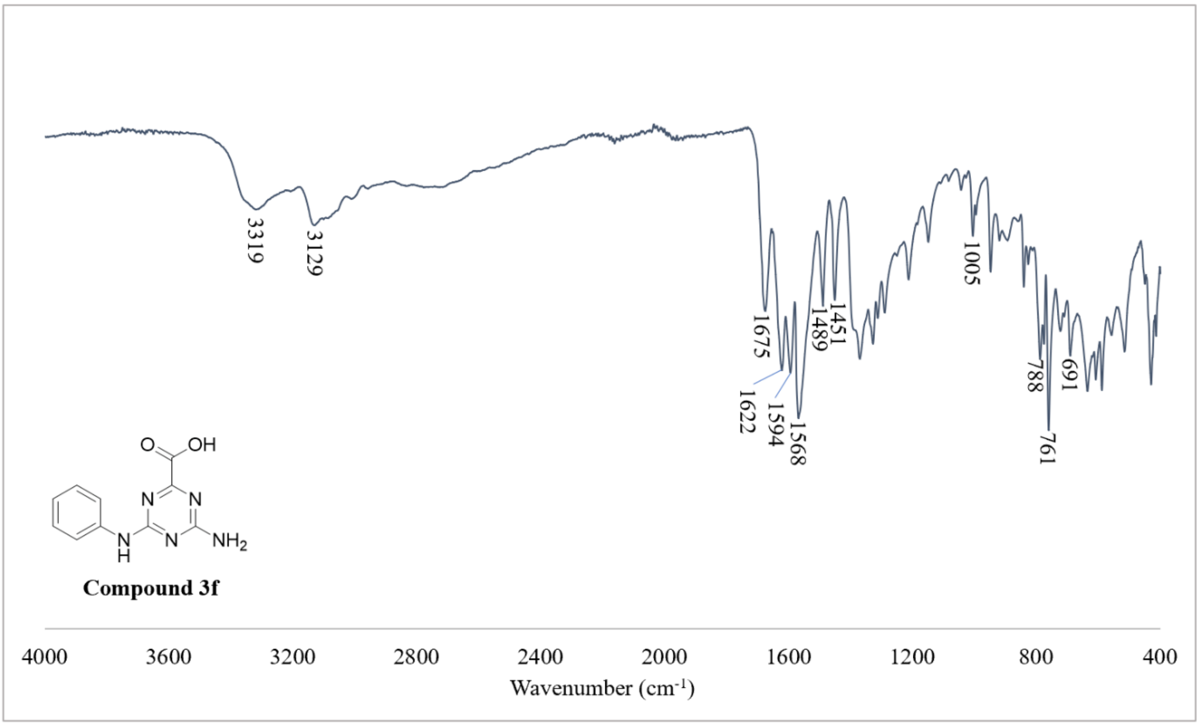

Figure 42. IR spectrum of 4-amino-6-(phenylamino)-1,3,5-triazine-2-carboxylic acid (**3f**)

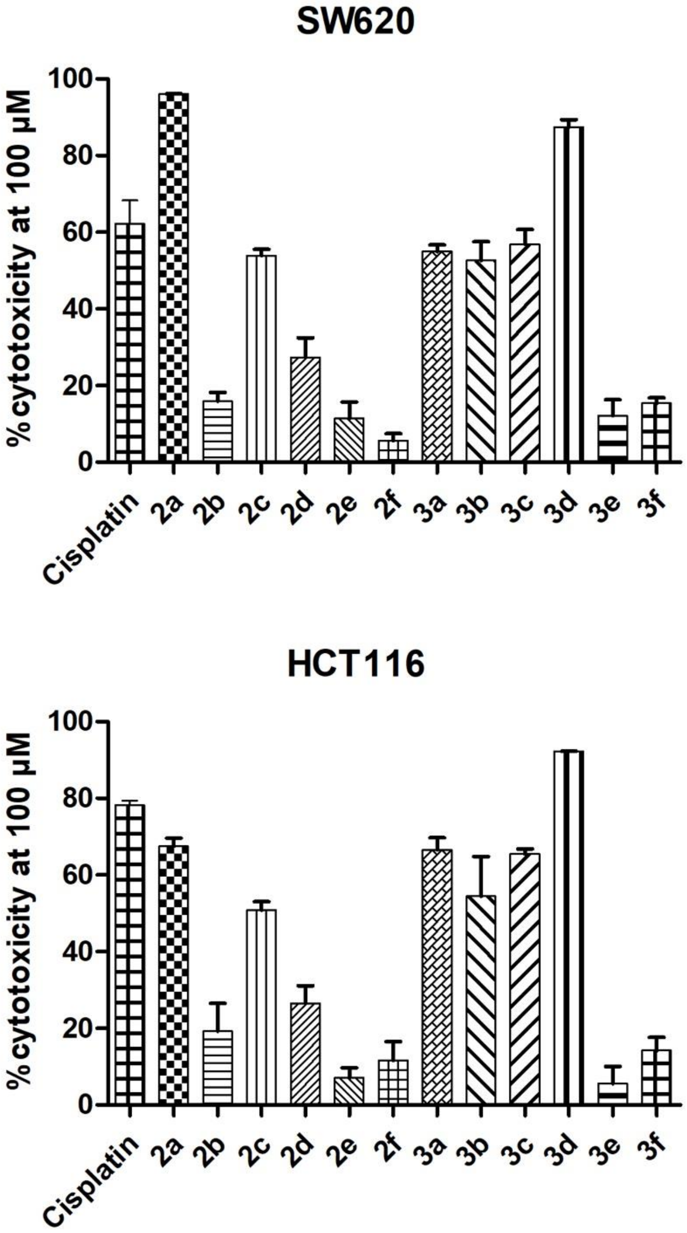

Figure 43. The cytotoxicity of twelve 1,3,5-triazine derivatives at 100 μM.

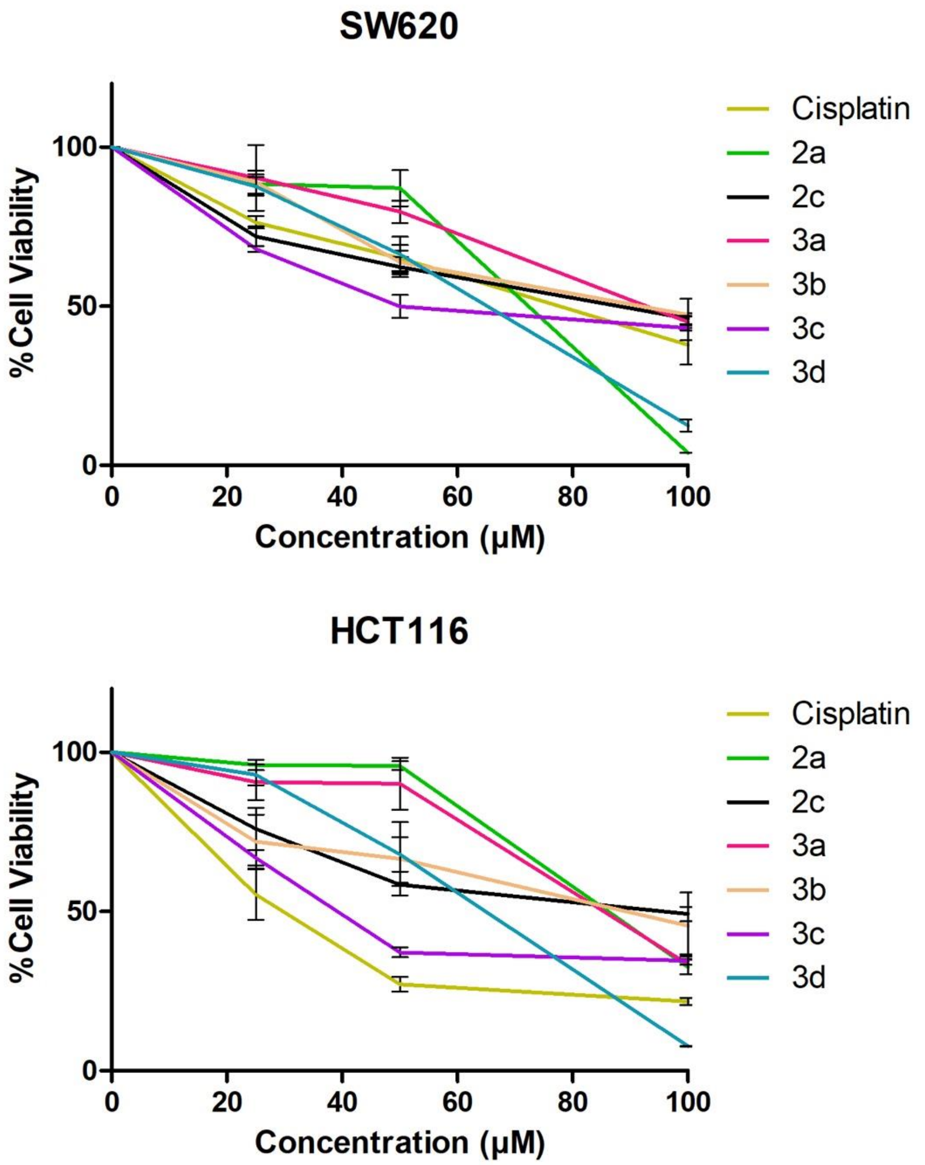

Figure 44. The concentration-response curves of the six 1,3,5-triazine derivatives at different concentrations (25, 50 and 100 µM) against SW620 and HCT116 cell lines for the calculation of IC_{50} values.

			Size (d.nm):	% Intensity:	St Dev (d.n...
Z-Average (d.nm):	369.3	Peak 1:	319.6	94.9	113.4
PdI:	0.464	Peak 2:	4908	5.1	701.4
Intercept:	1.01	Peak 3:	0.000	0.0	0.000
Result quality :	Refer to quality report				

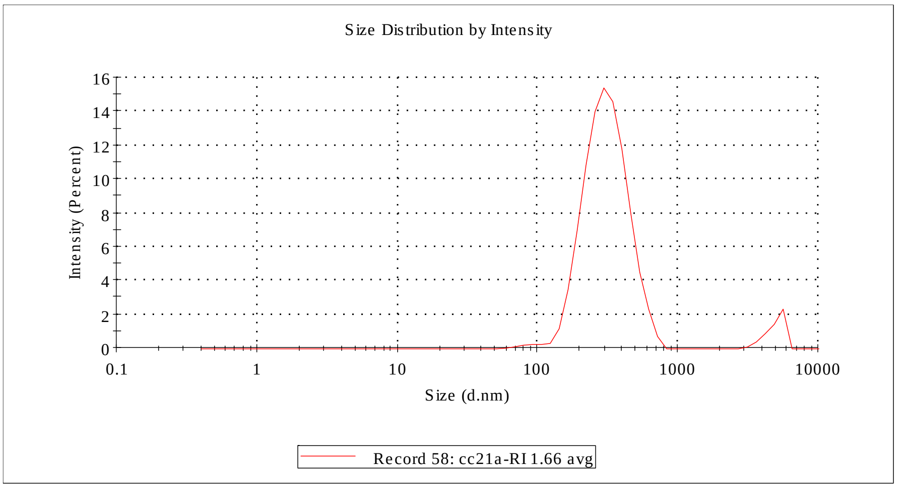

Figure 45. Particle size distribution determined by DLS – Condition A

			Size (d.nm):	% Intensity:	St Dev (d.n...
Z-Average (d.nm):	300.3	Peak 1:	326.3	94.7	140.4
PdI:	0.322	Peak 2:	4915	5.3	693.6
Intercept:	0.947	Peak 3:	0.000	0.0	0.000
Result quality :	Good				

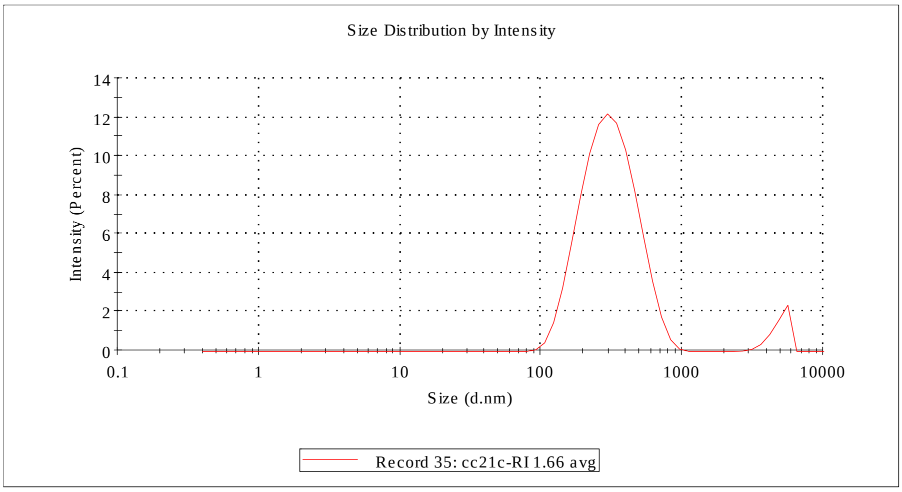

Figure 46. Particle size distribution determined by DLS – Condition C
